# SDE19, a SEC-dependent effector from ‘*Candidatus* Liberibacter asiaticus’ suppresses plant immunity and targets *Citrus sinensis* Sec12 to interfere with vesicle trafficking

**DOI:** 10.1371/journal.ppat.1012542

**Published:** 2024-09-10

**Authors:** Guiyan Huang, Xiaopeng Chang, Yanan Hu, Fuxuan Li, Nian Wang, Ruimin Li

**Affiliations:** 1 China-USA Citrus Huanglongbing Joint Laboratory, National Navel Orange Engineering Research Center, College of Life Sciences, Gannan Normal University, Ganzhou, China; 2 Jiangxi Provincial Key Laboratory of Pest and Disease Control of Featured Horticultural Plants, Gannan Normal University, Ganzhou, China; 3 Citrus Research and Education Center, Department of Microbiology and Cell Science, IFAS, University of Florida, Lake Alfred, Florida, United States of America; University of California, Davis Genome Center, UNITED STATES OF AMERICA

## Abstract

Citrus huanglongbing (HLB), which is caused by the phloem-colonizing bacteria *Candidatus* Liberibacter asiaticus (CLas), poses a significant threat to citrus production worldwide. The pathogenicity mechanism of HLB remains poorly understood. SEC-dependent effectors (SDEs) have been suggested to play critical roles in the interaction between citrus and CLas. Here, we explored the function of CLIBASIA_05320 (SDE19), a core SDE from CLas, and its interaction with its host target. Our data revealed that *SDE19* is expressed at higher level during infection of citrus than that during infection of the Asian citrus psyllid. Subcellular localization assays showed that SDE19 is localized in the nucleus and cytoplasm and is capable of moving from cell to cell in *Nicotiana benthamiana*. To investigate whether SDE19 facilitates pathogen infection, we generated transgenic *Arabidopsis thaliana* and citrus plants overexpressing SDE19. Transgenic *A*. *thaliana* and citrus plants were more susceptible to *Pseudomonas syringae* pv. *tomato* (*Pst*) and *Xanthomonas citri* subsp. *citri* (*Xcc*), respectively. In addition, RNA-seq analysis demonstrated that overexpression of *SDE19* resulted in a reprogramming of expression of genes related to biotic stimulus responses. SDE19 interacts with *Citrus sinensis* Sec12, a guanine nucleotide exchange factor responsible for the assembly of plant COPII (coat protein II)-coated vesicles, which mediate vesicle trafficking from the ER to the Golgi. SDE19 colocalizes with Sec12 in the ER by binding to its N-terminal catalytic region, affecting the stability of Sec12 through the 26S proteasome. This interaction hinders the secretion of apoplastic defense-related proteins such as PR1, P69B, GmGIP1, and RCR3. Furthermore, the secretion of PR1 and callose deposition is decreased in *SDE19*-transgenic *A*. *thaliana*. Taken together, SDE19 is a novel virulent SDE secreted by CLas that interacts with Sec12 to disrupt vesicle trafficking, inhibit defense-related proteins secretion, and promote bacterial infection. This study sheds light on how CLas manipulates the host vesicle trafficking pathway to suppress the secretion of defense-related proteins and interfere with plant immunity.

## Author summary

SEC-dependent effectors play a crucial role in the infection process of *Candidatus* Liberibacter asiaticus (CLas) in plants. However, the specific functions of most SDEs remain poorly understood. Here, we conducted functional analyses of SDE19, a core SDE, and revealed that it suppresses plant immunity and interferes with vesicle trafficking. We found that SDE19 is able to move intercellularly within plants and suppress the expression of defense-related genes and callose deposition to suppress plant immunity. Furthermore, overexpression of SDE19 enhances susceptibility of transgenic plants to bacterial infection. Specifically, SDE19 targets and destabilizes a citrus guanine nucleotide exchange factor, to interfere with host vesicle trafficking and inhibit the secretion of defense-related proteins. Our study revealed a new mechanism by which CLas suppresses plant immunity via interfering with vesicle trafficking.

## Introduction

Citrus huanglongbing (HLB), caused by *Candidatus* Liberibacter asiaticus (CLas), severely impacts the citrus industry worldwide [1,2]. Currently, there are no efficient HLB management strategies in HLB endemic regions [3]. CLas is a phloem-colonizing, obligate parasitic Gram-negative bacterium that cannot be cultured solely in vitro [4]. The genome size of the CLas is approximately 1.23 Mb and lacks type III (T3SS) and type IV secretion systems (T4SS), but possesses a complete type I secretion system (TISS) and the general secretory pathway (GSP / Sec translocon) [5,6]. Sec translocon (SEC) is a major protein translocation route for intracellular bacteria, and SEC-dependent effectors (SDEs) are of great importance for the pathogens like CLas and phytoplasmas to infect plants and insects [7,8].

In CLas, a total of 86 candidate SDEs were identified, with 36 exhibiting increased expression in citrus hosts and 8 in the Asian citrus psyllid (ACP) host [9]. Further clustering analysis identified 27 core SDEs that are conserved across various CLas strains [10]. Previous studies have revealed that multiple SDEs of CLas interact with different host targets to interfere with citrus immune responses. For example, SDE1 interacts with citrus papain-like cysteine protease, inhibiting citrus protease activity and suppressing plant immune response [11]. Overexpression of SDE1 in grapefruit increases susceptibility of citrus plants to CLas [12]. SDE15 inhibits plant immunity and promotes the colonization of CLas by interacting with citrus ACD2 [13]. SDE3 interacts with citrus 3-phosphoglyceraldehyde dehydrogenase GAPCs, regulates ATG8-mediated autophagy, and accelerates the proliferation of CLas in plants [14]. Furthermore, SDE4405 directly interacts with citrus ATG8, negatively regulating plant immune response. Overexpression of SDE4405 in sweet orange significantly enhances autophagic response [15]. Interestingly, many SDEs also interact with other CLas proteins [16]. In addition to SDEs, prophage-encoded effectors [17], nonclassical secreted effectors [18] and small peptide [19] may also be secreted into host plant cells to interfere with host cellular processes and plant immunity. The identification of many CLas effector proteins interfering with citrus immune response is consistent with the model that HLB is pathogen-triggered immune disease and the needs to mitigate the host immune responses for CLas survival inside the phloem [20]. However, many SDEs remain poorly characterized including CLIBASIA_05320 (SDE19).

Vesicle trafficking is a fundamental process for the transportation of proteins, lipids, and other molecules involved in endocytosis and secretion pathways [21]. It plays a significant role in the interaction between plants and pathogens as it is an important communication channel of plant cells, which transports or secretes defense-related substances to combat bacterial, fungal, and oomycete pathogens [22,23]. Consequently, the components of this pathway are frequently targeted by pathogens. High throughput screening of the interacting proteins of RXLR effectors from *Phytophthora infestans* revealed that a large number of RXLRs seemed to target vesicle trafficking machinery [24]. Specifically, PexRD12/31 was found to interact with NbVAMP72x, a component of secretory vesicles and the Golgi apparatus [24]. Another *P*. *infestans* effector, PexRD54, interacted with the small GTPase Rab8a, disrupting vesicle trafficking and utilizing lipid droplets to recruit autophagosomes to the site of pathogen invasion [25]. Additionally, the effector PsAvh181 of *P*. *sojae* inhibits the secretion of defense-related proteins GmGIP1, P69B, and PR1 by targeting soybean GmSNAP-1 [26]. Besides, barley powdery mildew pathogen effectors also target host ARF-GAP (ADP ribosylation factor-GTPase activating protein) to interfere with vesicle trafficking [27]. Furthermore, the effector HopM1 from *Pseudomonas syringae* interacts with AtMIN7, an adenosine diphosphate (ADP) ribosylation factor (ARF) guanine nucleotide exchange factor (GEF), leading to its degradation by the 26S proteasome, thus inhibiting plant immune response [28,29]. These findings highlight that plant vesicle trafficking-related proteins are important targets of pathogen effectors.

In this study, we conducted functional analyses of SDE19 (CLIBASIA_05320), a core SDE effector from CLas. SDE19 moves from cell to cells and interacts with a GEF to interfere with vesicle trafficking and reprograms the transcriptome of citrus. Our study provides new insights regarding how CLas manipulates plant vesicle trafficking and interferes with the secretion of defense-related proteins.

## Results

### SDE19 is highly expressed during infection of citrus and is able to move intercellularly in plant

SDE19 (CLIBASIA_05320) was previously identified as a secreted protein of CLas and was highly expressed in planta compared to in psyllids [9,30], and it was proposed to be one of the core SDEs conserved across various CLas strains [10]. SDE19 consists of 85 amino acids, with a predicted signal peptide (SP) of 22 amino acids at its N-terminal (S1A Fig). The secretion activity of the SP of SDE19 was evaluated by PhoA assay in the model bacteria *Escherichia coli*, confirming that the SP of SDE19 is able to direct PhoA to translocate outside of the bacterial cytoplasm via the Sec translocon (S1B Fig) [9]. To verify the expression profiles of *SDE19*, we conducted reverse transcription-quantitative PCR (RT-qPCR) analysis. The results showed that the expression of *SDE19* during infection of *Citrus sinensis* was 15 times higher than that in the corresponding ACP (S1C Fig), confirming the previous observation [30] and suggesting that it may participate in the progression of CLas infection of citrus plants.

**Fig 1:**
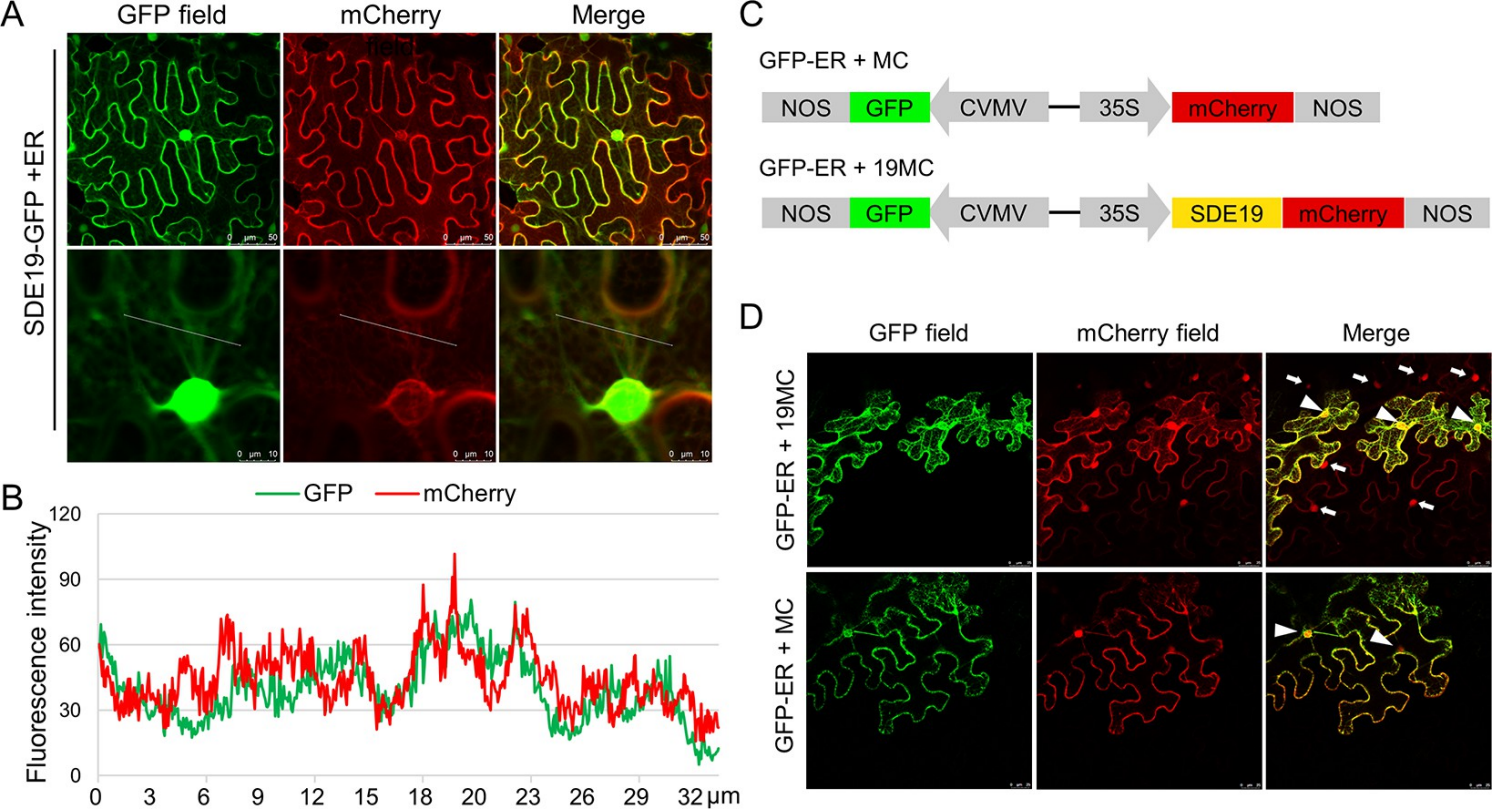
Subcellular localization and cell-to-cell movement analyses of SDE19-GFP. (A) Confocal images of SDE19-GFP and the endoplasmic reticulum marker. SDE19-GFP and ER-mCherry were co-expressed in *Nicotiana benthamiana* and observed at 2 days post-inoculation (dpi) of *Agrobacterium tumefaciens*. (B) A profile of the fluorescence intensities of GFP and mCherry aligned with the white line in (A). (C) The schematic diagram of expression construct for cell-to-cell movement experiments. (D) Confocal images show the cell-to-cell movement of SDE19-mCherry fusion proteins in *N*. *benthamiana* at 2 dpi. The infiltration was conducted at a final OD_600_ of 0.001 to enable single-cell transformation. The original transformed plant cells exhibiting both strong green and red fluorescence signals are marked with triangles. The cell-to-cell movement of SDE19-mCherry is indicated by the observation of mCherry but not GFP signal in cells surrounding the transformed cells, marked with white arrows.

The effectors secreted by pathogens often target diverse subcellular locations to interfere with host cell processes when expressed in *Nicotiana benthamiana* [31]. To determine the subcellular localization of SDE19, the mature sequence of *SDE19* without SP was inserted into the plant binary expression vector pCAMBIA2300-GFP. The SDE19-GFP fusion was then transiently expressed in *N*. *benthamiana* alone or with the endoplasmic reticulum (ER) marker [32]. The SDE19-GFP fusion showed both cytoplasmic and nuclear localization, with little colocalization with the ER marker (Figs 1A, 1B and S1D). For bacteria colonizing in sieve elements, their secreted effectors are likely to move to companion cells or other neighboring cells through the plasmodesmata. To assess whether SDE19 can migrate intercellularly, a vector with two expression frames featuring ER-localized GFP and mCherry was constructed, and SDE19 was fused with C-terminal mCherry (Fig 1C). After transient expression in plant cells, GFP-ER cannot move from cell to cell, so it labels the original transformed cells. The free mCherry showed co-localization with GFP, whereas the red fluorescence of SDE19-mCherry appeared not only in cells with GFP fluorescence, but also in surrounding cells without GFP fluorescence (Fig 1D). This indicates that SDE19 is capable of cell-to-cell movement. This is consistent with its small size of 9.5 kDa and plasmodesmata allows translocation of proteins less than 40 kDa [33].

### Overexpression of SDE19 reduces disease resistance

To investigate whether *SDE19* facilitates pathogen infection in plants, we obtained three transgenic *Arabidopsis thaliana* lines overexpressing *SDE19-GFP* and tested their resistance to *P*. *syringae* pv. *tomato* (*Pst*) DC3000. The *SDE19-GFP* transgenic *A*. *thaliana* lines were verified by semi-quantitative PCR and western blot (WB) (S2A and S2B Fig). The amount of bacterial colonization in the rosette leaves was measured at 3 days post-inoculation (dpi) of *Pst* DC3000. *Pst* colonization in *SDE19-GFP* transgenic *A*. *thaliana* lines was significantly higher than that in control plants (S2C Fig). Moreover, the expression levels of marker genes for salicylic acid (SA) and jasmonic acid (JA) biosynthesis and signaling pathway, including *ICS1*, *PAL1*, *PR1*, *LOX2* and *VSP2*, were significantly reduced in the *SDE19* transgenic plants at 36 hours post-inoculation (hpi) (S2 Fig). This suggests that overexpression of *SDE19* reduced the resistance of *A*. *thaliana* to *Pst* DC3000. In addition, the transgenic plants showed no difference in reactive oxygen species (ROS) production triggered by flg22, as demonstrated by DAB and NBT staining results (S3 Fig). In conclusion, *SDE19* reduced plant defense responses and facilitated pathogen infection in *A*. *thaliana*.

**Fig 2:**
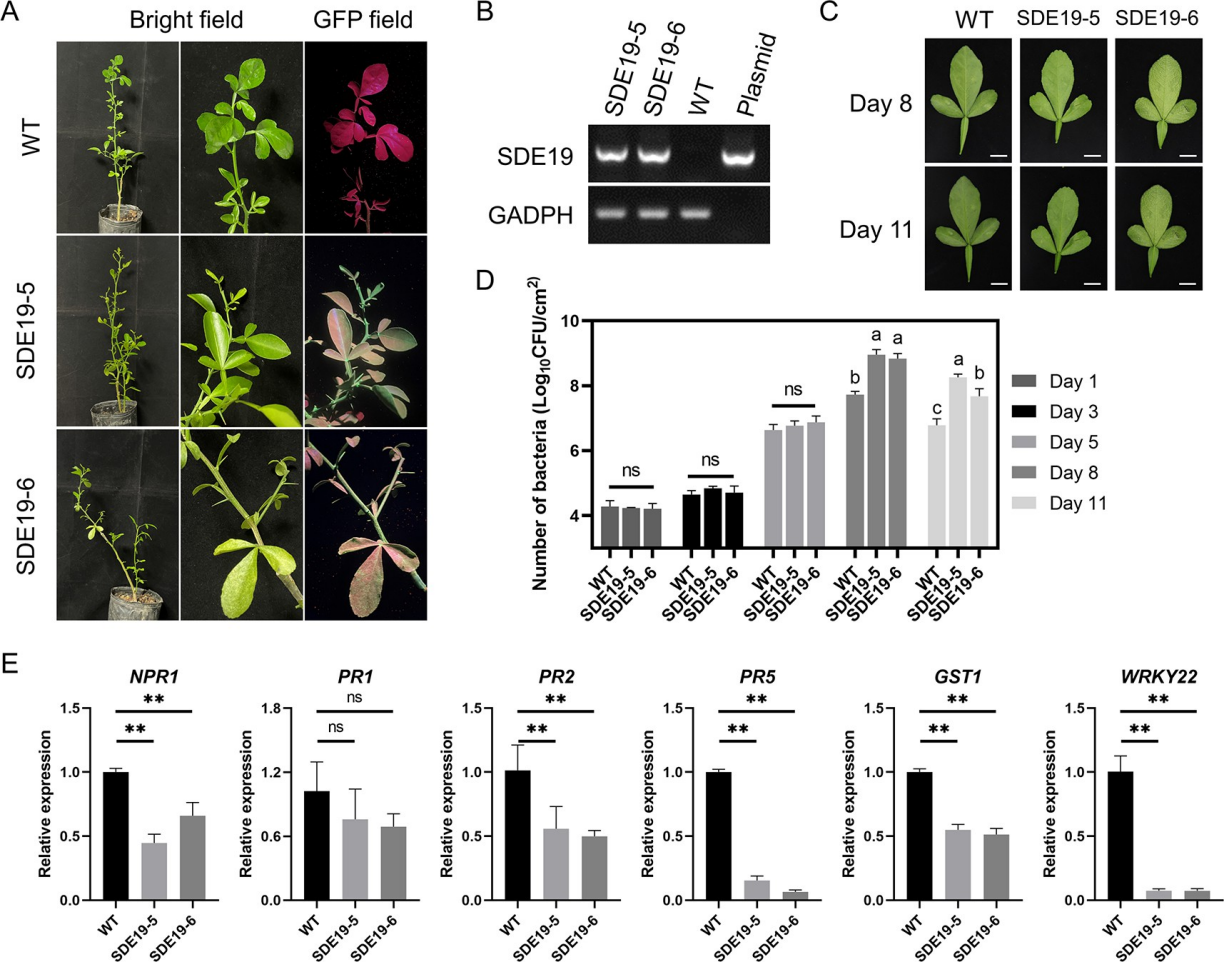
*SDE19* transgenic citrus lines exhibit enhanced susceptibility. (A) The phenotypes of *SDE19* transgenic citrus lines. Under blue light, transgenic plants displayed green fluorescence, whereas wild-type (WT) citrus emitted red fluorescence. (B) Analysis of the expression of *SDE19* in transgenic citrus lines by semi-quantitative PCR. (C) The symptoms of leaves from *SDE19* transgenic citrus lines and WT inoculated with *Xanthomonas citri* subsp. *citri* (*Xcc*) at 8 and 11 days post-inoculation (dpi). (D) Proliferation of *Xcc* was determined as colony forming units per cm^2^ (CFU/ cm^2^) at different stages post inoculation. Different letters (a, b, and c) above the bar indicate statistically significant differences (*P* < 0.05) based on a one-way ANOVA followed turkey’s multiple range test. ‘ns’ indicates no significant. (E) Analysis of the expression profiles of defense related genes in *SDE19* transgenic citrus plants at 2 dpi of *Xcc*. Relative expression of *NPR1*, *PR1*, *PR2*, *PR5*, GST1 and *WRKY22* genes was calibrated by internal reference gene *GAPDH*. Double asterisks indicate *P* value less than 0.01, while ns signifies a *P* value greater than 0.05 using Student’s *t* test.

To further investigate whether *SDE19* reduces disease resistance of the host plant, we generated two *SDE19* transgenic lines of ‘Carrizo’ citrange [*C*. *sinensis* (L.) Osb x *Poncirus trifoliata* (L.) Raf.], as evidenced by GFP fluorescence and semi-quantitative PCR (Fig 2A and 2B). *Xanthomonas citri* subsp. *Citri* (*Xcc*) was inoculated in the leaves of *SDE19* transgenic citrus lines and wild-type (WT) citrange, and the bacterial populations were continually measured at 1, 3, 5, 8, and 11 dpi. The results showed that *Xcc* growth at 8 and 11 dpi in *SDE19* transgenic citrus lines was significantly higher than that in WT (Fig 2C and 2D). Furthermore, the expression of defense-related genes, such as *NPR1*, *PR2*, *PR5*, *GST1* and *WRKY22*, was significantly reduced in *SDE19* transgenic citrus lines, while the expression of *PR1* remained unchanged at 2 days after *Xcc* inoculation (Fig 2E).

### *SDE19* reprograms the expression of biotic stimulus-related genes

**Fig 3:**
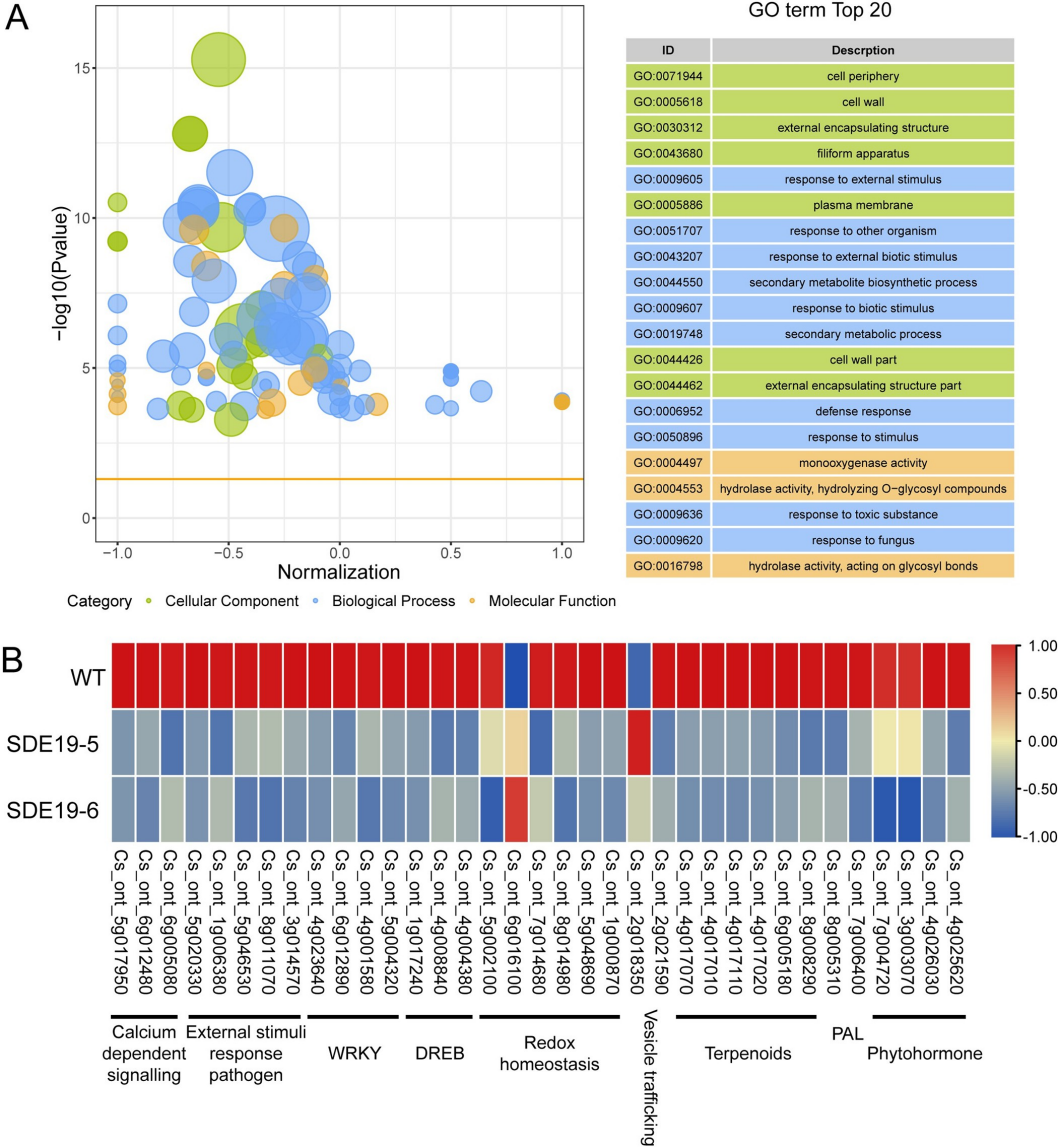
SDE19 reprogrammed the expression of biotic stimulus related genes in transgenic citrus plants. (A) GO enrichment analysis of the differentially expressed genes (DEGs) between *SDE19* transgenic citrus lines and wild-type citrus. (B) Heatmap of selected DEGs.

To gain insight into the effects of *SDE19* on biological process in transgenic citrus plants, we performed RNA-seq analysis and identified 963 DEGs, including 658 down regulated and 305 were up regulated (S4 Fig and S3 Table). We then conducted GO and KEGG enrichment analysis of DEGs between WT and *SDE19* transgenic citrus lines. GO enrichment analysis indicated a serious of GO terms associated with biotic stimulus including ‘response to external stimulus’, ‘response to external biotic stimulus’, ‘secondary metabolite biosynthetic process’, ‘response to biotic stimulus’, ‘secondary metabolic process’, ‘defense response’, ‘response to stimulus’, and ‘response to fungus’ were enriched (Fig 3A). Moreover, KEGG enrichment analysis demonstrated that ‘Phenylpropanoid biosynthesis’, ‘Plant-pathogen interaction’, ‘Sesquiterpenoid and triterpenoid biosynthesis’, and ‘MAPK signaling pathway’ were ranked in the top 20 enriched pathways (S5 Fig).

Functional annotation of the DEGs indicated that the expression profiles of various genes related to abiotic and biotic stresses were affected in the *SDE19* transgenic citrus lines (Fig 3B and S3 Table). Specifically, five DEGs involved in pathogen response, including *CBP60/SARD*, *SIB*, *EDS1*, *PAD4*, and *NLR*, were down regulated (Fig 3B). The expression of DREB and WRKY transcription factors, which regulate plants responses to biotic and abiotic stresses, was also reduced (Fig 3B). In addition, genes related to phytohormones such as indole-3-acetic acid dioxygenase, IAA/AUX, cytokinin dehydrogenase (CKX) and jasmonic acid transporter (JAT) were down-regulated in the transgenic plants (Fig 3B). Genes responsible for terpene biosynthesis, including phenylalanine ammonia lyase (*PAL*) genes, mono-/sesquiterpene-/diterpene synthase genes, lycopene beta cyclase gene and isoprenyl diphosphate synthase gene, showed decreased expression levels (Fig 3B). Interestingly, five of the six redox homeostasis related DEGs including two *AORs*, one *GST*, and two *RBOHs*, were also inhibited in *SDE19* transgenic citrus lines (Fig 3B). Three calcium sensor (*CML*) genes showed down-regulated expression (Fig 3B). Overall, the RNA-seq analysis demonstrated that the overexpression of *SDE19* led to a reprogramming of biotic stimulus-related gene expression, potentially leading to the enhanced susceptibility of the transgenic citrus.

### SDE19 interacts with *C*. *sinensis* Sec12

**Fig 4:**
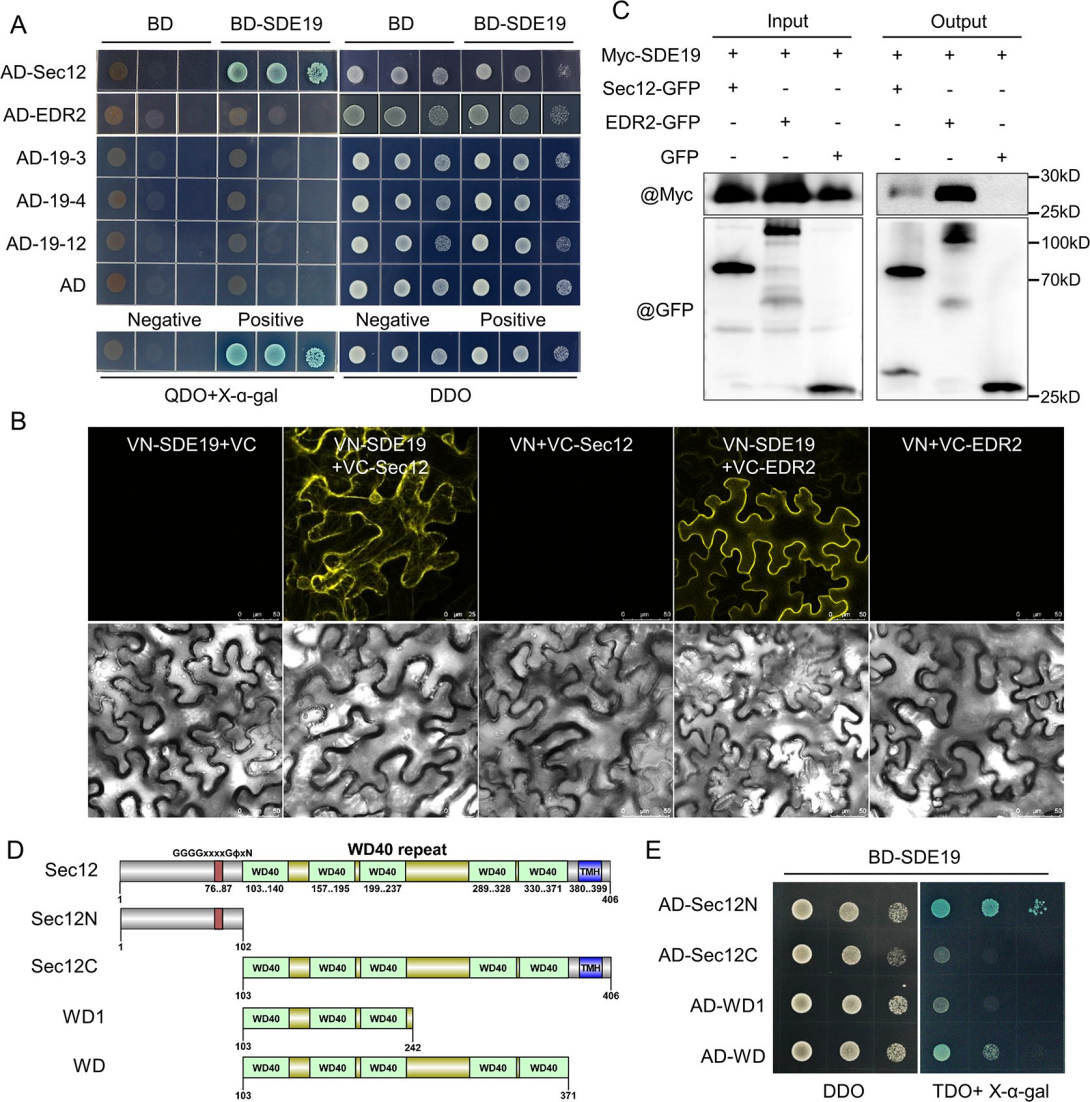
SDE19 interacts with Sec12 protein in *Citrus sinensis*. Verification of the interaction of SDE19 with candidate targets by yeast two-hybrid (Y2H) (A), bimolecular fluorescence complementation (BiFC) (B), and co-immunoprecipitation (Co-IP) (C). Constructing truncated mutants of Sec12 (D) to determine the core domains of Sec12 that interact with SDE19 by Y2H (E). (A and E) In the Y2H assay, *SDE19* was cloned into pGBKT7 (BD), the candidate target genes and truncated mutants of Sec12 were cloned into pGADT7 (AD) vector. They were co-transformed into yeast Y2Hgold strain, with the co-transformation of empty vectors as controls. Negative control: pGBKT7-Lam+pGADT7-T, positive control: pGBKT7-53+pGADT7-T. DDO: SD/-Leu/-Trp, QDO: SD/-Leu/-Trp/-His/-Ade, TDO: SD/-Leu/-Trp/-His. (B) In the BiFC assay, VN-SDE19 was constructed by fusing SDE19 with the N-terminal of Venus, while VC-Sec12 and VC-EDR2 were constructed by fusing candidate target genes with the C-terminal of Venus. VN-SDE19 was co-expressed with VC-Sec12 and VC-EDR2 in *Nicotiana benthamiana*, with the co-expression of empty vector as controls. The yellow fluorescence was observed at 2 days post-inoculation (dpi). Bars = 50 μm. (C) In the Co-IP assay, myc-tagged SDE19 was co-expressed with GFP-Sec12, GFP-EDR2 and GFP in *N*. *benthamiana* respectively. Total proteins were purified at 2 dpi and then incubated with GFP-Trap beads. The input and output proteins were detected by western blot with anti-myc and anti-GFP antibodies. (D) Diagram illustrating the protein structure of Sec12 and its truncated mutants. The protein structures were identified using SMART online (http://smart.embl-heidelberg.de/) with the default parameters. Sec12N: amino acids 1–102, Sec12C: amino acids 103–406, WD1: amino acids 103–242, and WD: amino acids 103–371.

To identify the host targets of SDE19 in *C*. *sinensis*, we conducted a yeast two-hybrid (Y2H) screening, resulting in the identification of five candidate targets (S4 Table). The interactions of the full-length candidates with SDE19 were confirmed using pairwise Y2H. The results showed that only *C*. *sinensis* Sec12 interacted with SDE19 in Y2H (Fig 4A). Sec12 is a GEF that interacts with the small GTP-binding protein to promote the exchange of GDP with GTP [34]. Subsequently, we employed bimolecular fluorescence complementation (BiFC) and co-immunoprecipitation (Co-IP) assay to further validate the interactions between SDE19 and Sec12, as well as another target, EDR2, an ENHANCED DISEASE RESISTANCE 2-like protein in *C*. *sinensis*. Interestingly, both candidate proteins interacted with SDE19 in BiFC and Co-IP assays (Fig 4B and 4C).

Sec12 family proteins have an N-terminal cytoplasmic domain with seven WD40 repeats, which possess GTP exchange activity and play a direct role in stabilizing COPII (coat protein II) coat assembly [35,36]. Prediction of the protein structure of Sec12 from *C*. *sinensis* using SMART indicated the presence of five WD40 repeats and a transmembrane helix (TMH) (Fig 4D). To identify the essential domains of Sec12 that bind to SDE19, we generated truncated mutants of Sec12 and analyzed their interactions using Y2H. The Y2H assay showed a strong interaction between SDE19 and Sec12N, while weaker interactions were observed with WD, WD1, and Sec12C (Fig 4E). A highly conserved motif of GGGGxxxxGϕxN is found in the Sec12N region, where G represents glycine, ϕ is a hydrophobic amino acid, and N is a strictly conserved asparagine essential for substrate binding and catalytic activity of Sec12 [35,36]. These findings indicate that SDE19 may interfere with the function of Sec12 by interacting with its N-terminal region.

### SDE19 colocalized with Sec12 in ER and reduced its protein stability

**Fig 5:**
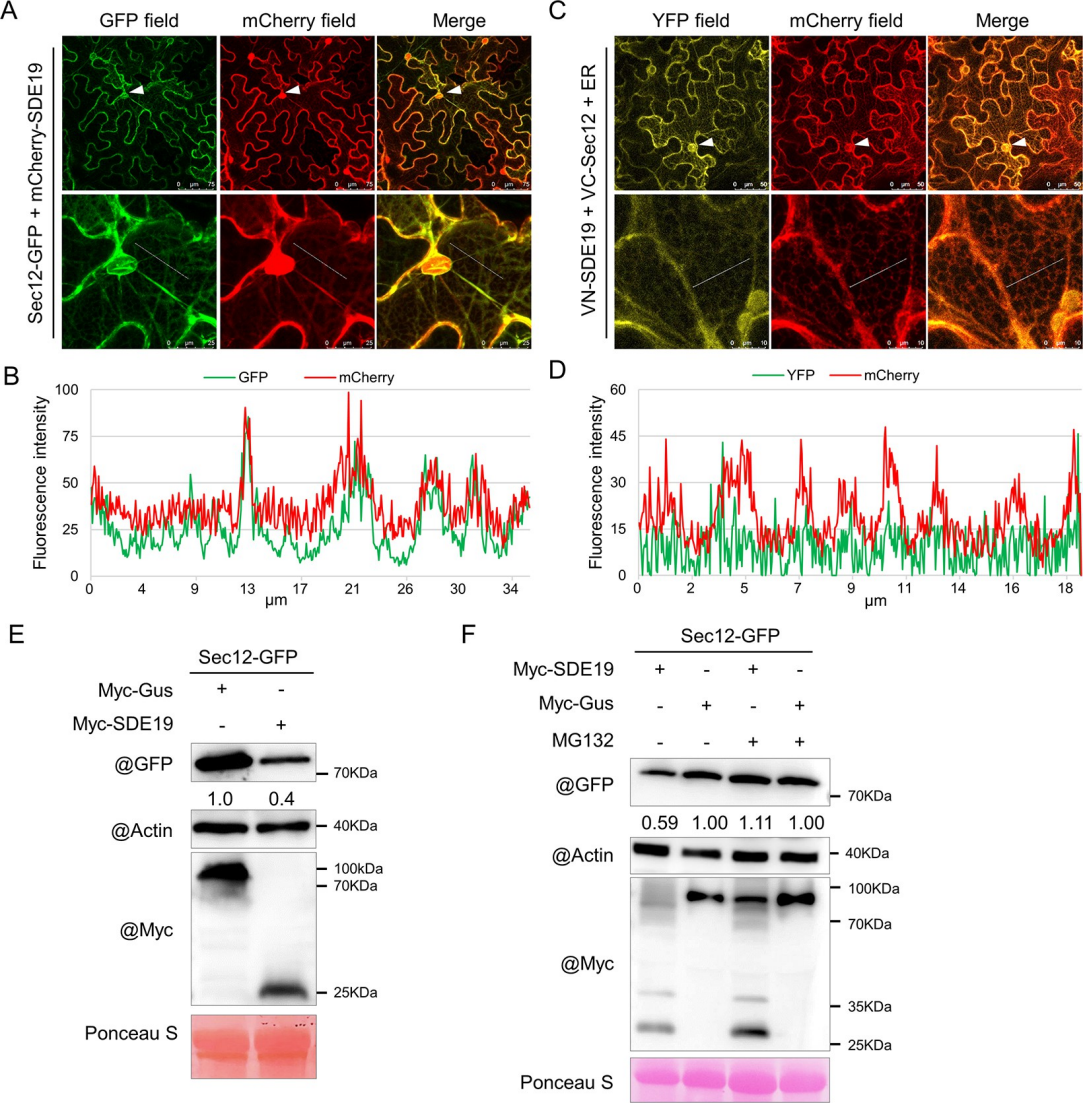
SDE19 colocalized with Sec12 in the endoplasmic reticulum (ER) and reduced its protein stability. (A) Sec12-GFP and mCherry-SDE19 were colocalized in the ER, but not in the nucleus. The bottom row displays partially enlarged micrographs of the ER network and cell nucleus. (B) A profile of the fluorescence intensities of GFP and mCherry aligned with the white line in (A). (C) VN-SDE19 and VC-Sec12 were co-expressed with ER marker in *Nicotiana benthamiana*. The bottom row displays partially enlarged micrographs of the ER network. (D) A profile of the fluorescence intensities of YFP and mCherry aligned with the white line in (B). The white triangles indicate the cell nucleus. (E) SDE19 reduced the protein stability of Sec12. Total proteins were extracted from *N*. *benthamiana* leaves co-expressing of Sec12-GFP and myc-SDE19 or myc-GUS at 2 days post-inoculation. (F) MG132 suppressed the destabilization of Sec12 triggered by SDE19. The leaves co-inoculated with Sec12-GFP and myc-SDE19 or myc-GUS were injected with 100 μM MG132 or its solvent DMSO solutions at 24 hours post-inoculation. The accumulation of Sec12-GFP was detected using anti-GFP antibodies, while actin levels were determined using anti-actin antibodies to indicate protein loading. The numbers listed under each band represent the relative abundance of Sec12-GFP, which has been normalized to actin.

Sec12 is a GEF localized in the ER which is involved in vesicle trafficking from the ER to the Golgi [37]. To confirm the localization of citrus Sec12, we co-expressed Sec12-GFP with an ER marker [32] in *N*. *benthamiana*. Confocal microscopy analysis showed that Sec12-GFP colocalized with the ER marker, indicating its ER localization (S6 Fig). When co-expressed with mCherry-SDE19, both proteins were localized inside ER, whereas the red fluorescence of mCherry-SDE19 was observed in the nucleus (Fig 5A and 5B). To verify the location of the interaction, the ER marker was further co-expressed with VN-SDE19 and VC-Sec12. The YFP fluorescence largely overlapped with the red fluorescence of the ER marker (Fig 5C and 5D), confirming the interaction between SDE19 and *C*. *sinensis* Sec12 occurred specifically within the ER.

Furthermore, we investigated the impact of *SDE19* on the expression of genes associated with vesicle trafficking, only two DEGs were identified in *SDE19* transgenic citrus, with gene encoding ESCRT-mediated sorting regulatory protein (*ISTL*) down regulated while gene encoding clathrin assembly protein (*AP180*) up regulated (Fig 3B). However, RNA-seq [38] and quantitative proteome analysis of HLB-positive *C*. *sinensis* showed that CLas infection resulted in the increased expression of *CsSAR1* and protein accumulation of CsSec13, CsSec16, CsSec23, and CsSec31 (S7 Fig). It’s worth noting that the accumulation of CsSec12 protein decreased during CLas infection (S7C Fig). To further confirm the impact of SDE19 on the protein stability of Sec12, we conducted a co-expression experiment in *N*. *benthamiana* and detected the protein accumulation using WB. Our results demonstrated a significant decrease in Sec12 protein accumulation when co-expressed with myc-SDE19 compared to the myc-GUS control. Meanwhile, the total protein and accumulation of actin remained consistent in both samples (Fig 5E), indicating that SDE19 effectively reduced the protein stability of Sec12, and potentially disrupted the balance of crucial components in vesicle trafficking.

To further clarify whether SDE19 destabilizes Sec12 through the 26S proteasome, *N*. *benthamiana* leaves co-expressing Sec12-GFP with myc-SDE19 or myc-GUS were treated with either the 26S proteasome inhibitor MG132 or its solvent dimethyl sulfoxide (DMSO). When treated with DMSO, the co-expression of SDE19 with Sec12 significantly reduced the accumulation of Sec12. However, when treated with MG132, the accumulation of Sec12 was not affected by SDE19 (Fig 5F). These results suggest that the interaction between SDE19 and Sec12 might facilitate its degradation through the 26S proteasome.

### SDE19 hinders the secretion of defense-related proteins

**Fig 6:**
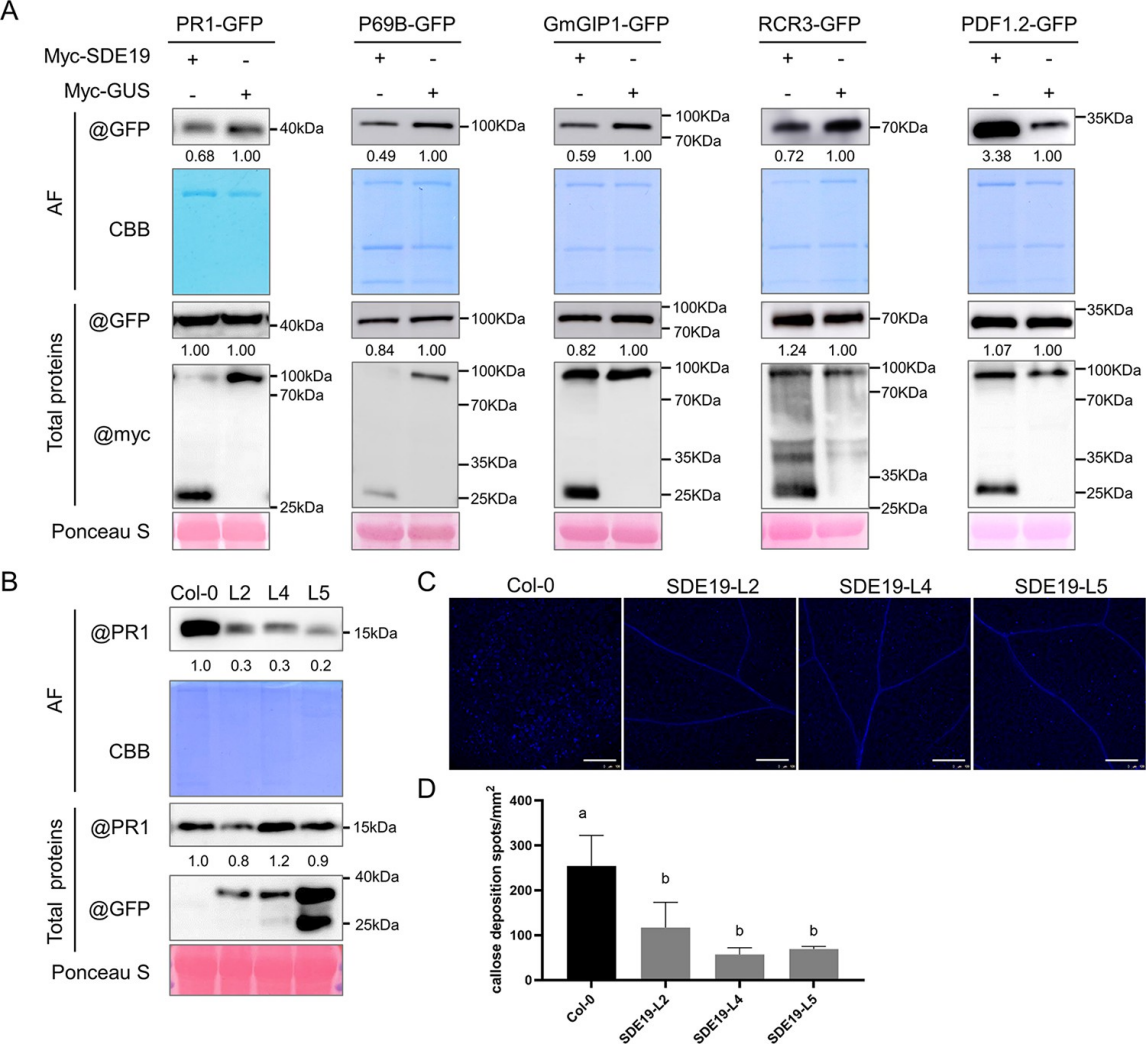
SDE19 inhibits the secretion of apoplastic defense-related proteins in plants. (A) PR1-GFP, P69B-GFP, GmGIP1-GFP, RCR3-GFP and PDF1.2-GFP were co-expressed with myc-SDE19 on one half of the *Nicotiana benthamiana* leaf, while they were co-expressed with myc-GUS on the other half, respectively. The apoplastic fluids (AFs) and total proteins were collected from each half of the leaf at 2 days post-inoculation. The levels of PR1-GFP, P69B-GFP, GmGIP1-GFP, RCR3-GFP and PDF1.2-GFP proteins were detected using anti-GFP antibodies. (B) The secretion of PR1 was disturbed in the *SDE19* transgenic *Arabidopsis thaliana*. The AFs and total proteins were collected from equal leaves of transgenic *A*. *thaliana* lines and wild-type Col-0 20 hours after spraying with 0.5 mM SA. The protein levels of PR1 were detected using anti-PR1 antibodies. (A-B) The numbers calculated by ImageJ represent the relative gray values of each band, indicating the relative abundance of apoplastic defense-related proteins. CBB indicated Coomassie brilliant blue staining of the AFs, while Ponceau S staining indicated the total protein loading. (C) Callose deposition was reduced in *SDE19* transgenic *A*. *thaliana*. The *A*. *thaliana* leaves were stained with aniline blue 20 hours after being infiltrated with 10 μM flg22, and photos were captured under ultraviolet light using a confocal microscope. Scale bar = 200 μm. (D) Statistical analysis of the callose deposits per mm^2^ were conducted using ImageJ. Different letters indicate statistically significant differences (*P* < 0.05) based on a one-way ANOVA followed by turkey’s multiple range test. Similar results were obtained from three independent experiments.

Sec12 plays a crucial role in the exchange of GDP with GTP in the Sar1-GDP complex, a process that is indispensable for the formation of COPII-coated vesicles and facilitates the transport of vesicles from ER to the Golgi (S7A Fig) [37,39]. We hypothesized that SDE19 may suppress the secretion of apoplastic defense-related proteins by interfering with the assembly of COPII-coated vesicles through destabilizing Sec12. To test this hypothesis, we examined the accumulation of pathogenesis-related protein 1 (PR1) [26], P69B [40], GmGIP1 [41], RCR3 [42] and PDF1.2 [43] in the apoplastic fluids (AFs) in the presence of SDE19. The full-length proteins tagged with C-terminal GFP, including PR1-GFP, P69B-GFP, GmGIP1-GFP, RCR3-GFP, and PDF1.2-GFP, were transiently co-expressed with myc-SDE19 on one half of the *N*. *benthamiana* leaf, while they were co-expressed with myc-GUS on the other half. The AFs and total proteins from each half of the leaf were collected and analyzed using WB. The results showed that, except for PDF1.2-GFP, the accumulation of PR1-GFP, P69B-GFP, GmGIP1-GFP, and RCR3-GFP proteins in the AFs was significantly reduced when co-expressed with myc-SDE19, compared to myc-GUS. Meanwhile, the levels of these defense-related proteins in the total proteins remained consistent in both the control and experimental groups (Fig 6A). Altogether, these findings indicated that SDE19 suppresses the secretion of defense-related proteins through interacting with and destabilizing Sec12.

To further confirm the influence of SDE19 on PR1 secretion, the secretion of PR1 induced by SA in the *SDE19* transgenic *A*. *thaliana* was monitored using anti-PR1 antibodies. The results showed that there was no significant variation in the level of PR1 in total proteins, however, its accumulation in AFs of three transgenic *A*. *thaliana* lines decreased significantly (Fig 6B). Furthermore, callose deposition in the transgenic *A*. *thaliana* was also examined, as it requires an intact vesicle trafficking pathway for the transport of callose synthase and serves as an indicator of plant immune response [44,45]. Two-week-old *A*. *thaliana* seedlings were stained with aniline blue 20 hours after being infiltrated with 10 μM flg22. The results showed a significant reduction in callose deposition in the leaves of the transgenic *A*. *thaliana* compared to the WT Col-0 (Fig 6C and 6D). Taken together, these findings demonstrated that the vesicle trafficking of defense-related proteins was disrupted in the *SDE19* transgenic *A*. *thaliana*.

### Silencing of *NbSec12* seriously affect the growth of *N*. *benthamiana*

**Fig 7:**
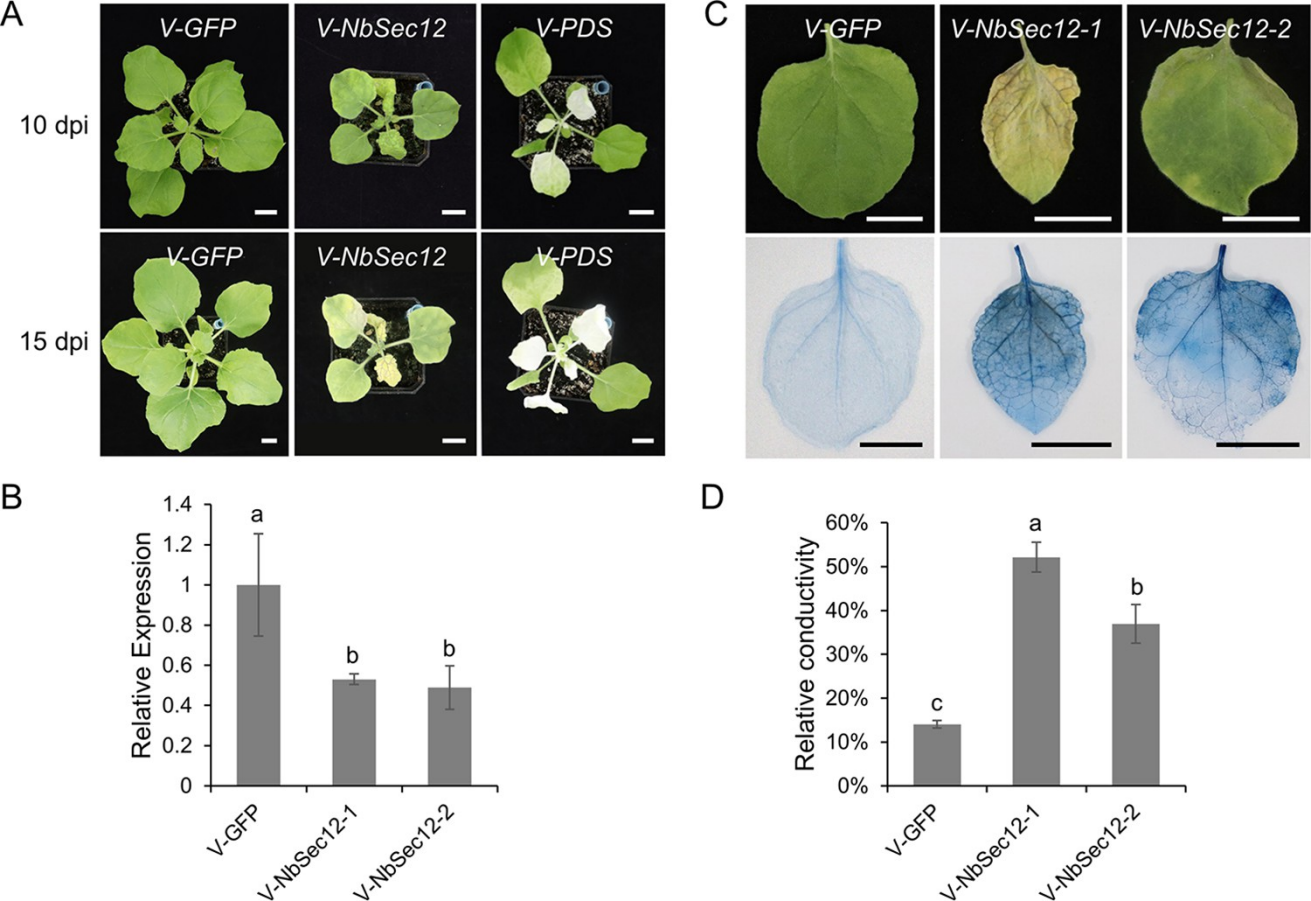
Silencing of *Sec12* seriously inhibits the growth of *Nicotiana benthamiana*. (A) The morphology of *N*. *benthamiana* plants treated with TRV::*GFP*, TRV::*NbSec12* and TRV::*PDS* at 10- and 15 days post-inoculation (dpi). (B) The relative expression profiles of *NbSec12* in *N*. *benthamiana* treated with TRV constructs at 10 dpi. *V-NbSec12-1* and *V-NbSec12-2* represent the upper and lower leaves in *NbSec12* silenced plant. *NbEF1α* was used as internal reference gene in the reverse transcription-quantitative PCR. Different letters indicate statistically significant differences (*P* < 0.05) based on a one-way ANOVA followed by turkey’s multiple range test. (C) Cell death of *N*. *benthamiana* leaves stained by trypan blue at 15 dpi. Scale bar = 2 cm. (D) The electrolyte leakage of *NbSec12*-silenced and control *N*. *benthamiana* leaves at 15 dpi. Different letters indicate statistically significant differences (*P* < 0.05) based on a one-way ANOVA followed by turkey’s multiple range test.

To investigate whether secretion of PR1 depends on Sec12, *N*. *benthamiana* was subjected to *Sec12* silencing using virus-induced gene silencing (VIGS) technology. The VIGS results revealed that silencing of *NbSec12* seriously affect the growth of *N*. *benthamiana* (Fig 7). By 10 dpi, the plants treated with TRV2::*NbSec12* were smaller than those treated with TRV2::*GFP* control. They exhibited stunted growth and noticeable shrinking in the apical leaves. When most of the *PDS*-silenced leaves exhibited chlorosis at 15 dpi, a large portion of the apical leaves in *NbSec12-*silenced plants had undergone cell death, resulting in the dieback of the apical bud (Fig 7A). The expression of *NbSec12* in both upper and lower leaves of *NbSec12*-silenced plants decreased to 50% of the control levels, as determined by RT-qPCR at 10 dpi (Fig 7B). Trypan blue staining and electrolyte leakage assays were conducted at 15 dpi to monitor the degree of cell death in both the upper and lower leaves in *NbSec12*-silenced plants. The results showed serious cell death in the upper leaves of *NbSec12*-silenced plants, particularly along the veins, indicative of severe cell membrane damage (Fig 7C and 7D). Since silencing of Sec12 leads to leaf cell death, it is challenging to directly examine PR1 or other protein secretion on it. The growth of the yeast *sec12-1* mutant is also hindered and results in lethality at 35°C, and *A*. *thaliana Sec12* is able to rescue the thermo-sensitivity and secretion phenotype of the yeast *sec12-1* mutant [46]. The silencing of Sec12 may result in the obstruction of COPII vesicle-mediated ER-to-Golgi vesicle transport, disrupting the delivery of proteins and substances that rely on this pathway. This silencing of Sec12 has significant consequences for plant growth, highlighting the essential role of Sec12 in the survival of plant cells.

## Discussion

**Fig 8:**
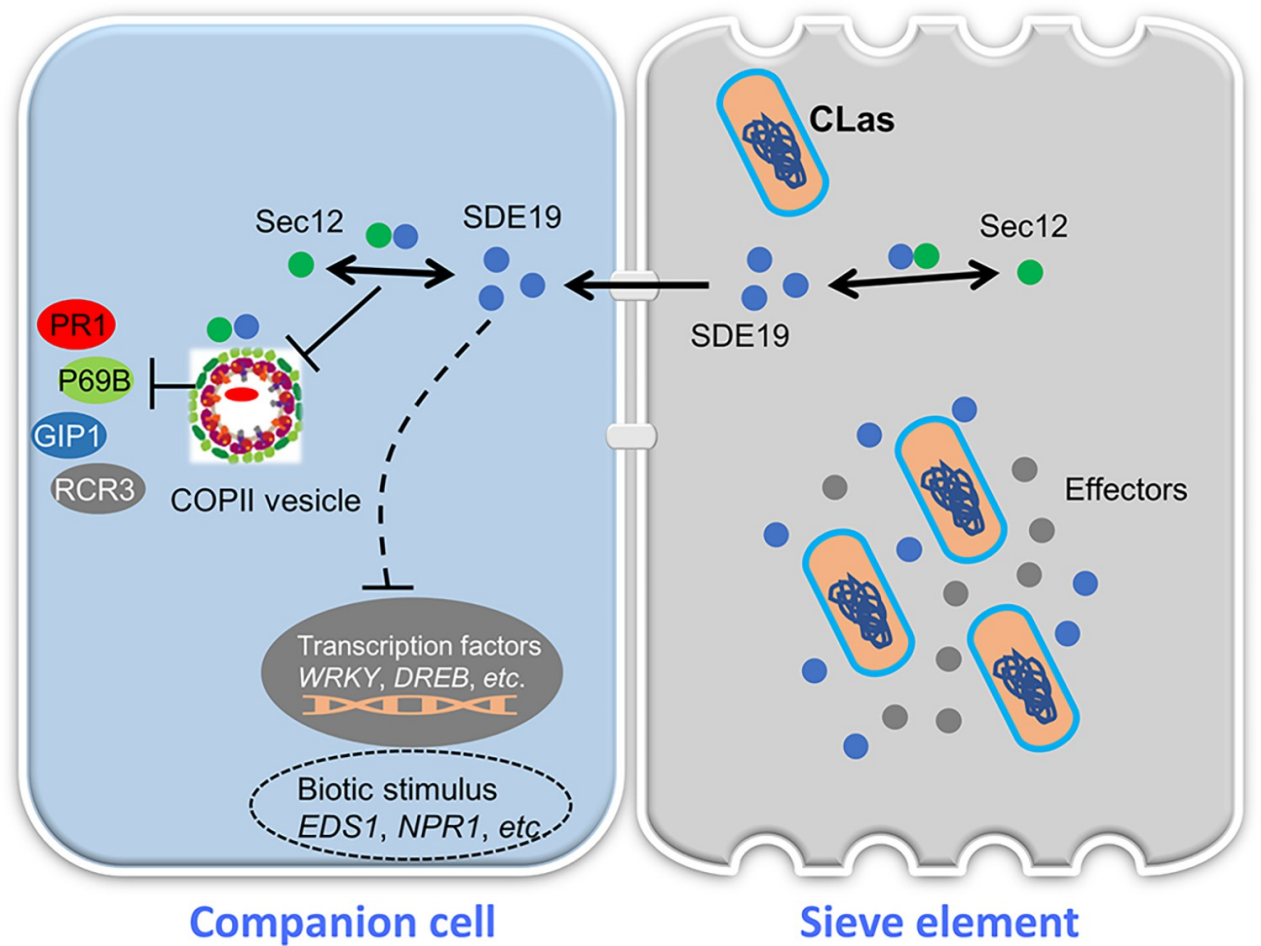
SDE19 disrupts vesicle trafficking by interfering with Sec12. A proposed model illustrating that SDE19 interacts with and destabilizes Sec12 to disturb vesicle trafficking and inhibit PR1 secretion. SDE19 is released by CLas into the host sieve element (SE), and then it moves from the SE to companion cells and neighboring cells through plasmodesmata. SDE19 reprograms the expression of genes related to biotic stimuli, including genes involved in redox homeostasis, phytohormone metabolic pathways, and various transcription factors, ultimately enhancing the susceptibility of host plants. Importantly, SDE19 interacts with and destabilizes Sec12 to perturb vesicle trafficking and inhibit secretion of defense-related proteins such as PR1, P69B, GmGIP1, and RCR3, in order to counteract plant resistance.

The study of the pathogenesis of CLas is severely limited due to its colonization in plant sieve elements and inability to conduct genetic manipulations. Recent studies have focused on the virulence functions of effector proteins secreted by CLas, revealing that SDEs such as SDE1, SDE15, SDE3, CLIBASIA_04425 and SDE4405 can suppress plant immunity and facilitate the colonization of pathogenic bacteria via targeting various citrus proteins [11,13–15,47]. In addition to SDEs, CLas also encodes a SA hydroxylase that degrades SA to suppress plant defense [48]. Among the 27 core SDEs predicted in CLas [10], the specific functions of most of them remain unknown. In this study, we demonstrate that SDE19 enhances bacterial infection by interacting with and destabilizing Sec12 to disrupt vesicle trafficking, and reprograming the transcriptome of citrus, representing a novel strategy employed by CLas to interact with the citrus plant (Fig 8).

As an intracellular bacterium, CLas is thought to secrete proteins through various pathways including the Sec translocon, TISS, OMVs, and other atypical secretion pathways [9,10,17,49]. Just like Phytoplasma, another phloem-colonizing bacterial pathogen, CLas adopt SDEs as virulence factors to attack plant during infection [7,10]. Here, we validated the secretion properties of SDE19 using the PhoA assay in the model bacterium *E*. *coli*, consistent with the previous study [9]. Once secreted into the sieve elements, SDE19 probably moves systemically through sieve pores. Furthermore, we found that SDE19 moves from cell-to-cell in *N*. *benthamiana* leaves (Fig 1). The size exclusion limits (SELs) of plasmodesmata between sieve cells and companion cells allow translocation of proteins < 40 kDa [7,33]. It is likely SDE19 exerts its effects inside the companion cells as CLas has dramatic impacts on sieve element and companion cells, but has relatively minor effects on other surrounding parenchyma cells [20]. Importantly, companion cells are critical for the function of sieve elements.

Multiple CLas SDEs have been shown to mitigate plant immune response via affecting different components of the plant immunity including affecting autophagy, ROS scavenging- related protein and papain-like cysteine proteases [12,14,15,17]. In this study, we have demonstrated that CLas can mitigate immune responses by interacting with *C*. *sinensis* Sec12 in the ER. Specifically, Sec12 is a GEF known to interact with the small GTP-binding protein Sar1, facilitating the formation of Sar1-GTP [50]. This leads to the subsequent assembly of the Sar1-GTP/Sec23/Sec24 complex (S7A Fig), which completes the assembly of COPII-coated vesicles [51]. During the vesicular transport process from the ER to the Golgi, the formation of Sar1-GTP catalyzed by Sec12 is essential for the budding of COPII vesicles [34]. Sec12 plays a key role in mediating vesicle trafficking from the ER to the Golgi and is a crucial component of protein sorting and transport in the secretory pathway. It is reported that vesicle trafficking mediated secretion of defense-related molecules is important for plant resistance to pathogens. On the other hand, pathogens have the capability to disrupt this process by releasing effectors to block the secretion of defense-related molecules such as PR1, secondary metabolites, and cell wall materials [23,29]. For instance, effectors from fungi, bacterial or oomycete pathogens target key components of the plant vesicle trafficking pathway including ARF-GAP [18], GTPases [52,53] and SNAREs [26,54] to disturb host secretion [24]. Moreover, *P*. *syringae* effector HopM1 interacted with AtMIN7, an ARF GEF, and mediated its degradation via the host proteasome to suppress plant immunity [28]. Here, we have shown that SDE19 specifically targets and destabilizes Sec12 through 26S proteasome to disrupt secretion of defense-related proteins. CLas inhabits in sieve cells, the inhibition of PR1 secretion may not directly influence its colonization. However, PR1 is a well-known marker for SA-induced plant immunity triggering systemic acquired resistance (SAR) upon secretion and cleavage [55,56]. Therefore, blocking the secretion of PR1, apoplastic proteases and hydrolase inhibitor may compromise plant SAR.

In addition to the well-known biological process of COPII-coated vesicles, recent researches have unveiled new roles for Sec12 and COPII-coated vesicles. The interaction between TMED9 and SEC12 facilitates the formation of membrane contact between the ER-Golgi intermediate compartment and the ER-exit site, which is crucial for promoting autophagosome biogenesis induced by various stress stimuli [39]. Despite the lack of reports in plants, the function of Sec12 in the assembly of COPII-coated vesicles is conserved across eukaryotes. Therefore, it is reasonable to speculate that Sec12 may be involved in the biogenesis of autophagosomes in response to stress in plants. Both SDE3 and SDE4405 of CLas have been shown to manipulate host autophagy to interfere with plant immunity [57]. While there is currently no evidence suggesting a direct link between SDE19 and autophagy, it is worth investigating how SDE19 may affect the autophagy process by interacting with Sec12. Moreover, it was reported that Sec12 and COPII-coated vesicles are involved in the transport of the nucleus-encoded proteins to the chloroplast [57], indicating the versatile functions of these components. By destabilizing Sec12, SDE19 potentially not only inhibits the secretion of apoplastic defense-related proteins but also impacts the transport of other plasma membrane proteins, chloroplast proteins or substances that depend on COPII-coated vesicles. Since the vesicle transport from the ER to Golgi mediated by Sec12 plays a crucial role in multiple cellular processes, it is not surprising that the silencing of Sec12 results in extensive cell death and significantly affects plant growth. In addition, silencing of genes involved in vesicle trafficking pathways also affects plant growth. For example, silencing of *NbSNAPs* and *Sec5*, the targets of PsAvh181 and AVR1, reduces plant growth [26,58]. CLas infection significantly reduces the accumulation of Sec12 protein in citrus (S7C Fig), which may contribute to distinct HLB symptoms such as systemic cell death of phloem cells, twig dieback, stunted growth of seedlings, and root decay.

Interestingly, SDE19 negatively affects plant immunity in both *A*. *thaliana* and citrus plants, suggesting it affects conserved components of immune response. Overexpression of SDE19 reduces callose deposition induced by flg22, which suggests that SDE19 may affect the transportation of callose synthase to the cell membrane via disrupts vesicular trafficking from the ER to the Golgi, and ultimately inhibiting callose deposition. This is similar to a previous report that *P*. *infestans* AVR1 interacts with Sec5 to disturb vesicle trafficking and callose deposition [58]. Another example is that RXLR242 interferes with RAB proteins, thereby disrupting the trafficking of the flg22 receptor FLAGELLIN-SENSING 2 to block downstream immunity [53]. SDE19 does not inhibit flg22-triggered ROS, suggesting that it suppresses plant immunity by disrupting protein secretion rather than affecting FLS2 internalization. In addition to its interaction with Sec12 in the ER, SDE19 was also located in the nucleus and reprogrammed the expression of citrus biotic stimulus related genes. These genes are known to be involved in SA signaling [59–61], redox homeostasis, and terpene biosynthesis [20,62], which are affected by CLas infection. Thus, it is probable that SDE19 may interact with other targets to interfere with plant immunity.

In sum, we demonstrate that SDE19, a core SDE effector of CLas, targets and destabilizes *C*. *sinensis* Sec12, a GEF, to interfere with host vesicle trafficking and inhibit the secretion of defense-related proteins. SDE19 functions as a virulence factor that suppresses plant immunity and promotes bacterial infection. This is the first study to reveal that CLas disrupts host vesicle transport pathways to interfere with plant immunity. Although SDEs from CLas target different citrus proteins, their role in disrupting host cell biological processes and suppressing plant immunity is consistent. This highlights the range of strategies CLas employs to attack host plants, posing new challenges for citrus resistance breeding.

## Materials and methods

### Microbial strains and plant materials

*E*. *coli* Top10 and BL21 (Weidibio Co., Ltd.) were cultured in Luria-Bertani medium with applicable antibiotics for vector construction. *Saccharomyces cerevisiae* strain Y187 and Y2Hgold (Weidibio Co., Ltd.) were used for yeast two-hybrid (Y2H). *Agrobacterium tumefaciens* GV3101 and EHA105 (Weidibio Co., Ltd.) were cultured in YEP medium and used for transient expression in *N*. *benthamiana* and genetic transformation of ‘Carrizo’ citrange. *Xcc* was cultured in nutrient broth (Difco, Detroit) (NB) or nutrient agar (NA) plates at 28°C. *N*. *benthamiana* and *A*. *thaliana* were grown in an artificial climate room with a 16/8 h light/dark photoperiod at 22°C, while citrus plants were grown in an artificial climate room with a 14/10 h light/dark photoperiod at 28°C. Asian citrus psyllids (ACP) *Diaphorina citri* were feeding on CLas-infected *C*. *reticulata* cv. Unshiu in fly nets in our laboratory, and CLas infected ACP along with the midribs of the infected *C*. *reticulata* were collected for RNA extraction.

### Plasmid construction

The vectors used in this study were listed in S1 Table and the primers were listed in S2 Table. In brief, all plasmids were constructed using homologous recombination methods with *pEASY*-Basic Seamless Cloning and Assembly Kit (TransGen Biotech). All constructed vectors were verified by Sanger sequencing (Tsingke Biotechnology).

### Alkaline phosphatase (PhoA) assay

PhoA assay was conducted following previously reported methods [9,63]. *PhoA* was cloned into vector pET28a with or without its signal peptide (SP) to construct pET28a-PhoA and pET28a-mPhoA. Then, the SP of SDE19 was fused in-frame with mphoA to generate pET28a-19SP-mPhoA. The resulting plasmids were transformed into the *E*. *coli* DH5α competent cells, and monoclonal colonies were identified by PCR. Then the positive *E*. *coli* cells were cultured on solid LB medium containing 50 μg/mL kanamycin, 90 μg/mL 5-bromo-4-chloro-3-indolyl-phosphate (BCIP), 75 mM Na_2_HPO_4_ and 1 mM isopropyl-b-D-thiogalactopyranoside (IPTG). The plates were cultured at 37°C for 16 hours followed by observation.

### RNA isolation and RT-qPCR analysis

Total RNA of ACP was isolated using TaKaRa MiniBEST Universal RNA Extraction Kit (TaKaRa) and total RNA of plant samples were isolated using plant RNA isolation kit (TransGen Biotech). Total RNA was reverse transcription using EasyScript All-in-One First-Strand cDNA Synthesis SuperMix for qPCR (TransGen Biotech). Quantitative PCR assays were conducted with TransStart Top Green qPCR SuperMix (TransGen Biotech). Relative expression values were calculated using 2^-ΔΔCt^ method [64] with DNA gyrase subunit B (gyrB) of *C*. Liberibacter asiaticus, *NbEF1α* of *N*. *benthamiana*, *glyceraldehyde-3-phosphate dehydrogenase (GAPDH)* of citrange as reference genes [13].

### *A*. *tumefaciens*-mediated transient expression and fluorescence observation

*A*. *tumefaciens* GV3101 carrying plant binary vectors were cultured, centrifuged, and then resuspended in MES buffer [10 mM 2-(N-morpholine)-ethane sulfonic acid (MES), 10 mM MgCl_2_, pH 5.6, and 200 μM acetosyringone [65]. *A*. *tumefaciens* resuspensions were injected into *N*. *benthamiana* leaves at a final OD_600_ value of 0.4 unless otherwise stated. For bimolecular fluorescence complementation (BiFC) [66], *A*. *tumefaciens* GV3101 carrying pDEST-VN and pDEST-VC derivatives were mixed in a 1:1 ratio at the final OD_600_ value of 0.4. Fluorescence was observed at 2 dpi using a laser scanning confocal microscope (Leica TCS SP8), with 488 nm excitation and 500–530 nm emission spectrum for GFP and YFP, and 559 nm excitation and 595–625 nm emission spectrum for mCherry.

### Genetic transformation of *A*. *thaliana* and bacteria inoculation assay

The inflorescences of *A*. *thaliana* WT Col-0 plants were dipped in *A*. *tumefaciens* GV3101 suspension containing the pCAMBIA2300-SDE19-GFP plasmid as described previously [65]. The transgenic *A*. *thaliana* lines were confirmed by semi-quantitative PCR. Rosette leaves from 4- to 5-week-old homozygous T3 plants and WT were then infiltrated with *Pst* DC3000 at a concentration of 10^5^ colony-forming units (CFU)/mL. At 3 dpi, sixteen leaves were collected from 8 plants, with each pair of leaves being immediately ground in 1 mL of distilled water, diluted serially, and then dripped onto King’s broth plates with 25 μg/mL rifampicin. Bacterial colonization was calculated as CFU/leaf and represented on a logarithmic scale [67]. These experiments were repeated three times with similar results.

### Callose deposition assay and ROS staining

Callose deposition assay was carried out following the previously reported method [68]. Three 2-week-old *A*. *thaliana* seedlings from each line were vacuum infiltrated with 10 μM flg22 (Ezbiolab). After 20 hours, the seedlings were treated with 95% ethanol at 37°C to remove the chlorophyll. The cleared seedlings were then washed in a series of ethanol/water solutions (70%, 50%, 30%) and rehydrated with distilled water. Subsequently, the seedlings were stained with 0.1% (w/v) aniline blue in 150 mM K_2_HPO_4_ (pH 9.5/KOH) in the dark for 30 minutes. The stained seedling leaves were mounted with 50% glycerol and captured under a confocal microscope (Leica TCS SP8) using ultraviolet light. The number of callose deposits in each image from 3 leaves were counted using ImageJ software [69]. To assess ROS accumulation, 2-week-old seedlings were treated with 10 μM flg22 for 3 hours, then stained with 0.5 mg/mL NBT (Coolaber) and 1 mg/mL DAB (DAB Color Development Kit, Coolaber) respectively [70]. The samples were then decolorized and imaged. Three independent experiments were performed with similar results.

### Genetic transformation of citrus

Genetic transformation of citrus was performed according to previous method [71]. *A*. *tumefaciens* EHA105 carrying pCAMBIA1380-GFP-SDE19 plasmid was used for citrus transformation. Seeds of ‘Carrizo’ citrange were surface sterilized and planted in MS medium to generate seedlings. The epicotyls of the seedlings were cut into pieces and co-incubated with *A*. *tumefaciens* resuspension solutions, then incubated in selection medium for shoots regeneration. Positive transgenic shoots were selected by GFP fluorescent observation and subsequently confirmed by semi-quantitative PCR. Following confirmation, these transgenic shoots were then grafted onto 2-year-old citrange seedlings for further analysis.

### Pathogen infection and evaluation of disease resistance of transgenic citrus

Citrus leaves from both the *SDE19*-transgenic and WT citrange were infiltrated with *Xcc* solutions, following the method described previously [13]. The *Xcc* strains were cultured overnight at 28°C, then centrifuged and resuspended in sterile water at a concentration of 10^6^ CFU/mL. Disease symptoms were noted and bacterial growth was measured at 1, 3, 5, 8, and 11 dpi. Furthermore, the inoculated leaves were collected at 2 dpi for the expression assay of defense-related genes. These experiments were carried out with three leaves from each transgenic line and were replicated three times with similar results.

### RNA-seq analysis

Three independent WT citrange plants and two *SDE19*-transgenic citrange plants were employed for RNA-seq analysis. Three leaves from each plant were pooled for RNA extraction using plant RNA isolation kit (TransGen Biotech) and the integrity of RNA were determined using Agilent 2100 bioanalyzer (Agilent). The sequencing libraries were constructed using NEBNext Ultra RNA Library Prep Kit for Illumina (NEB). The insert size of sequencing libraries was determined using Agilent 2100 bioanalyzer (Agilent) and cDNA libraries were sequenced using an Illumina NovaSeq 6000 platform. The raw sequencing data was filtered by Trimmomatic v 0.39 [72] and mapped to reference genome of *C*. *sinensis* v3.0 [73] using Hisat2 [74]. The mapping ratio ranged from 86.76% to 88.10%. Specifically, the mapping ratios for the three WT citrange plants were 87.78%, 88.10%, and 87.48% respectively. For SDE19-5 and SDE19-6, the ratios were 86.76% and 87.58% respectively. Then StringTie 1.3.3b [75] was used to estimate the expression level of transcripts, and featureCounts 1.5.0 [76] was used to count the reads number of each gene for calculating the FPKM value of each gene. DEseq2 was used to identify differentially expressed genes (DEGs) with a threshold of |log2FC| ≥1 and FDR < 0.05. OmicShare online tools (www.omicshare.com) were employed to conduct Gene ontology and KEGG enrichment of DEGs, and MapMan was used for functional annotation [77].

### Yeast two-hybrid library screening

To identify the target proteins in citrus, we performed Y2H library screening with SDE19 as a bait. The citrus cDNA library used for Y2H was constructed in previous study [78]. pGBKT7 (BD)-SDE19 was transformed into Y2Hgold competent cells and the transcription activation abilities of SDE19 were evaluated. Then Y2Hgold [BD-SDE19] and citrus cDNA library cells were co-cultured and plated onto triple dropout medium SD/-Leu/-Trp/-His (TDO). The grown monoclonal colonies were diluted on quadruple dropout medium SD/-Leu/-Trp/-His/-Ade (QDO) with 40 μg/mL X-α-gal, and the blue colonies were selected for PCR amplification and sequencing. The sequencing results were analyzed by BLAST in the NCBI database to generate the full-length coding sequences of the candidate targets of SDE19 in *C*. *sinensis*. These coding sequences were then cloned and inserted into pGADT7. The generated plasmids were co-transformed with BD-SDE19 into Y2Hgold followed by transferred onto SD/-Leu/-Trp medium, TDO and QDO with 40 μg/mL X-α-gal to evaluate the positive interactions. Interactions were demonstrated by growth and blue color of yeast transformants on TDO or QDO with 40 μg/mL X-α-gal after 3–5 days of culture [13].

### Co-immunoprecipitation and Western blot

Myc-SDE19 was co-expressed with Sec12-GFP, EDR2-GFP, or free GFP in *N*. *benthamiana* leaves via *A*. *tumefaciens*-mediated transient expression. Subsequently, total proteins were extracted with RIPA buffer (Solarbio), and precipitated with GFP-Trap agarose beads (Proteintech) following the product manual. The precipitates were washed at least six times with wash buffer containing 0.1% NP-40. Immunoblotting was then carried out to detect both the total proteins (input) and the precipitated proteins (output) using anti-myc or anti-GFP antibodies as described previously [79].

In protein stability assays, Sec12-GFP was co-expressed with myc-SDE19 or myc-GUS on each half of the *N*. *benthamiana* leaf. The total proteins from each half of the leaf were extracted at 2 dpi for WB assay. MG132 treatment was carried out as described previously [80]. The leaves were injected with 100 μM MG132 or its solvent DMSO solutions at 24 hpi and were harvested for protein extraction at 48 hpi. Following the WB analysis, the grayscale values of Sec12-GFP and actin bands were measured using Image J. The relative level of Sec12-GFP protein was calculated by comparing the grayscale values of the Sec12-GFP band to the actin band, and then normalizing this ratio to the control group. These experiments were conducted in at least three leaves from different plants with similar results.

For western blot, proteins were separated using SDS-PAGE electrophoresis and transferred to PVDF membranes. Subsequently, the PVDF membranes were blocked with skimmed milk and incubated with primary antibodies overnight at 4°C. After being washed four times with TBS-T buffer, The PVDF membranes were incubated with secondary antibodies for 2 hours. Finally, the proteins were identified using the eECL Western Blot Kit (CWBIO) after three washings with TBS-T buffer and one with TBS buffer.

### Apoplastic fluids collection

After co-transient expression with *A*. *tumefaciens* GV3101 carrying part27-myc-SDE19 and pCAMBIA2300-PR1-GFP derivatives or part27-myc-GUS and pCAMBIA2300-PR1-GFP derivatives in *N*. *benthamiana* leaves, the apoplastic fluids (AFs) were collected using vacuum filtration with ice-cold water. The infiltrated leaves were dried on the surface and centrifuged for 5 min at 1000 g in a 20 mL syringe placed in a clean 50 mL centrifuge tube [81]. The AFs were then mixed with 1 x protein loading buffer (TransGen Biotech) and boiled for subsequent WB analysis.

### Virus-induced gene silencing in *N*. *benthamiana*

The silencing fragment of NbSec12, designed using the SGN VIGS tool (https://vigs.solgenomics.net), was incorporated into the TRV2 vector through homologous recombination. The resulting TRV2 vector was subsequently introduced into *A*. *tumefaciens* GV3101. To achieve infiltration, *A*. *tumefaciens* carrying TRV2::*NbSec12* or TRV2::*GFP* was combined in a 1:1 ratio with *A*. *tumefaciens* carrying TRV1, with TRV2::*PDS* used to monitor the silencing process. The concentration of each strain was adjusted to OD_600_ of 0.25. Subsequently, the mixed bacteria suspension was injected into the lower leaf of four-leaf stage *N*. *benthamiana* plants [65]. Cell death was observed in *N*. *benthamiana* leaf through trypan blue staining when *NbSec12* was silenced.

### Electrolyte leakage determination

Equal amount of *N*. *benthamiana* leaves treated with TRV::*GFP* or TRV::*NbSec12* were immersed in 5 mL of deionized water and left at 25°C for 30 min. The conductivity E1 was then measured using a DDS-11A conductivity meter (Leici, China). The samples were then boiled for 10 minutes, and the conductivity E2 was measured again using the conductivity meter. The relative electrolyte leakage was calculated using the formula E1/E2*100% as previous described [82].

### Statistical analysis

SPSS v 25.0 (IBM) was used for statistical analysis. Turkey’s multiple range test or Student’s *t* test was applied to the data, and *P* value less than 0.05 was considered as significant and *P* value less than 0.01 was considered as very significant.

## Supporting information

S1 Data Excel spreadsheet containing, in separate sheets, the underlying numerical data and statistical analysis for Figure panels 1B, 2D, 2E, 3A, 3B, 5B, 5D, 6D, 7B, 7D, S1C, S2C, S2D-2M, S4, S5, and S6B. (XLSX)

S1 Table The vectors used in this study. (DOCX)

S2 Table Primers used in this study. (DOCX)

S3 Table Differential expressed genes between *SDE19* transgenic citrus lines with wild-type plants. (XLSX)

S4 Table Candidate targets of SDE19 screened by yeast two-hybrid. (XLSX)

S1 Fig The secretion and expression analyses of SDE19 from CLas. (A) Sequence analysis of SDE19, (B) The signal peptide of SDE19 is capable of directing protein secretion. The signal peptide of SDE19 was fused with mature PhoA to assess alkaline phosphatase activity in *Escherichia coli*. Full length *phoA* was used as the positive control, while mature PhoA without SP served as the negative control for non-secretion. (C) Relative expression of *SDE19* in CLas-infected *Citrus sinensis* and Asian citrus psyllids by reverse transcription-quantitative PCR (RT-qPCR). *gyrB* was used as an endogenous control. Bars represent the standard deviation (SD) of the means, double asterisks indicate *P* value less than 0.01 using Student’s *t* test. (D) Subcellular localization analysis of SDE19-GFP in *Nicotiana benthamiana*.
(TIF)

S2 Fig *SDE19* enhances the susceptibility of transgenic *Arabidopsis thaliana*. (A) Growth phenotype of *SDE19* transgenic *A*. *thaliana* lines. (B) Verification of *SDE19* expression using semi-quantitative PCR and western blot. *AtUBC9* was used as the internal reference gene. The asterisks (*) represent the protein bands of SDE19-GFP, which were detected by anti-GFP antibodies. (C) *SDE19* promotes the colonization of *Pseudomonas syringae* pv. *tomato* (*Pst*) strain DC3000 in transgenic *A*. *thaliana* plants. Mature leaves of transgenic *A*. *thaliana* lines and wild-type Col-0 were inoculated with *Pst* DC3000 cell suspensions. Bacterial colonization was determined as colony forming units (CFU/ leaf) at 3 days post-inoculation. Bars represent the standard deviation (SD) of the means from 8 samples of 16 leaves. Different letters (a, b, and c) above the bar indicate statistically significant differences (*P* < 0.05) based on a one-way ANOVA followed by turkey’s multiple range test. Similar results were obtained from three independent experiments. (D-M) The relative expression of *ACS2*, *ACS6*, *EIN2*, *ERF6*, *ICS1*, *LOX2*, *PAL1*, *PDF1*.*2*, *PR1* and *VSP2*, in *SDE19* transgenic *A*. *thaliana* at 36 hours post-inoculation of *Pseudomonas syringae* pv. *tomato* DC3000 was detected by reverse transcription-quantitative PCR. Bars represent the standard deviation (SD) of the means, an asterisk indicates *P* value less than 0.05, and double asterisks indicate *P* value less than 0.01 using Student’s *t* test. *AtUBC9* was used as the internal reference gene.
(TIF)

S3 Fig ROS staining results of *SDE19* transgenic *Arabidopsis thaliana*. (A) DAB staining of hydrogen peroxide (H_2_O_2_) accumulation in *SDE19* transgenic *A*. *thaliana* and wild-type Col-0. (B) NBT staining of superoxide in *SDE19* transgenic *A*. *thaliana* and Col-0.
(TIF)

S4 Fig Volcano plot analysis of differentially expressed genes between *SDE19* transgenic citrus lines and wild-type plants. The blue number represents the quantity of down-regulated genes, while the red number represents the quantity of up-regulated genes.
(TIF)

S5 Fig KEGG enrichment analysis of differentially expressed genes between *SDE19* transgenic citrus lines and wild-type plants. (TIF)

S6 Fig Sec12-GFP was colocalized with the ER marker in *Nicotiana benthamiana*. (A) Confocal images of Sec12-GFP and the mCherry labeled endoplasmic reticulum marker were taken at 2 days post-inoculation. The bottom row shows partially enlarged micrographs of the ER network. (B) A profile of the fluorescence intensities of GFP and mCherry aligned with the white line in (A).
(TIF)

S7 Fig The impact of CLas infection on the assembly process of host COPII-coated vesicles. (A) Schematic diagram of the COPII-coated vesicles assembly process adapted from [36]. (B) RNA-seq analysis revealed differential expressed genes involved in the vesicle trafficking pathway during CLas infection. SARA represents SECRETION-ASSOCIATED RAS 1. (C) Quantitative proteome analysis revealed differential accumulation of proteins involved in the vesicle trafficking pathway during CLas infection. The red dots represent up-regulated genes or proteins, while the blue dots represent down-regulated proteins. FC stands for Fold change, while #N/A indicates that the protein was not detected during mass spectrum analysis.
(TIF)
