# Supplementary material for: SDE19, a SEC-dependent effector from ‘Candidatus Liberibacter asiaticus’ suppresses plant immunity and targets Citrus sinensis Sec12 to interfere with vesicle trafficking
Source: PLoS Pathog. 2024 Sep 10;20(9):e1012542. doi: 10.1371/journal.ppat.1012542 (PMC11414923; doi:10.1371/journal.ppat.1012542)
Supplement: S1 Table — (DOCX) [file ppat.1012542.s002.docx]

Table S1. The vectors used in this study

| List | Vector | Usage | Note |
| --- | --- | --- | --- |
| 1 | pET28a-19SP-mPhoA | Alkaline phosphatase activity |  |
| 2 | pET28a-PhoA |  |  |
| 3 | pET28a-mPhoA |  |  |
| 4 | pCAMBIA2300-GFP | Subcellular localization and cell-to-cell movement |  |
| 5 | pCAMBIA2300-SDE19-GFP |  |  |
| 6 | pCAMBIA1380-GFP-mCherry |  |  |
| 7 | pCAMBIA1380-GFP-SDE19-mCherry |  |  |
| 8 | pGBKT7 | Yeast two hybrid |  |
| 9 | pGBKT7-SDE19 |  |  |
| 10 | pGADT7 |  |  |
| 11 | pGADT7-CsSec12 |  | XM_006469118 |
| 12 | pGADT7-CsEDR2 |  | XM_006473892 |
| 13 | pGADT7-19-3 |  | XM_025099614 |
| 14 | pGADT7-19-4 |  | XM_006489870 |
| 15 | pGADT7-19-12 |  | XM_006492691 |
| 16 | pGADT7-Sec12N |  |  |
| 17 | pGADT7-Sec12C |  |  |
| 18 | pGADT7-WD1 |  |  |
| 19 | pGADT7-WD2 |  |  |
| 20 | pGBKT7-p53 |  |  |
| 21 | pGADT7-T |  |  |
| 22 | pGBKT7-Lam |  |  |
| 23 | pDEST-VN | Bimolecular fluorescence complementation |  |
| 24 | pDEST-VN-SDE19 |  |  |
| 25 | pDEST-VC |  |  |
| 26 | pDEST-VC-CsSec12 |  |  |
| 27 | pDEST-VC-CsEDR2 |  |  |
| 28 | part27-Myc-SDE19 | Protein stability and inhibition secretion of defense-related proteins |  |
| 29 | part27-Myc-GUS |  |  |
| 30 | pCAMBIA2300-CsSec12-GFP |  |  |
| 31 | pCAMBIA2300-PR1-GFP |  |  |
| 32 | pCAMBIA2300-P69B-GFP |  |  |
| 33 | pCAMBIA2300-GmGIP1-GFP |  |  |
| 34 | pCAMBIA2300-RCR3-GFP |  |  |
| 35 | pCAMBIA2300-PDF1.2-GFP |  |  |
| 36 | pCAMBIA1380-SDE19 | Citrange genetic transformation |  |
| 37 | TRV2-NbSec12 | Virus induced gene silence |  |
| 38 | TRV2-GFP |  |  |
| 39 | TRV2-PDS |  |  |
