# Supplementary material for: SDE19, a SEC-dependent effector from ‘Candidatus Liberibacter asiaticus’ suppresses plant immunity and targets Citrus sinensis Sec12 to interfere with vesicle trafficking
Source: PLoS Pathog. 2024 Sep 10;20(9):e1012542. doi: 10.1371/journal.ppat.1012542 (PMC11414923; doi:10.1371/journal.ppat.1012542)
Supplement: S2 Table — (DOCX) [file ppat.1012542.s003.docx]

Table S2. Primers used in this study

| Primer name | Sequences |
| --- | --- |
| QSDE19-F | CGGAACAGAAGAGAAAGGAGAC |
| QSDE19-R | GGTGTTTGTTTAGTCGCATTAGG |
| gyrB-F | GTATGGCACAGGACTGGTCT |
| gyrB-R | GTTAGGGCGGAAATCAACAGT |
| phoA-F | ACTTTAAGAAGGAGATATACCATGgtgaaacaaagcactattgcact |
| phoA-R | TGGTGGTGGTGGTGGTGCTCGAGtttcagccccagagcggctt |
| mphoA-F | TTTAAGAAGGAGATATACATGGGATCCGCCAAGCTTcggacaccagaaatgcctgttc |
| mphoA-R | TGGTGGTGGTGGTGGTGCTCGAGtttcagccccagagcggctt |
| GFP-SDE19-F | AGAACACGGGGGACGAGCTCATGGCCAATGAGCACTCTTCTGT |
| GFP-SDE19-R | ACCATGGTGTCGACTCTAGAATTGTTTAAGCCTCCAAAGAGC |
| 1380MC-19-F | AACACGGGGGACTGGTACCATGGCCAATGAGCACTCTTCTGT |
| GFP-SDE19-R | ACCATGGTGTCGACTCTAGAATTGTTTAAGCCTCCAAAGAGC |
| 1380-SDE19-F | GGAGAGAACACGGGGGACTCTAGAATGGCCAATGAGCACTCTTCTGT |
| 1380-SDE19-R | gtcgtatgggtaGTCGACGGTACCATTGTTTAAGCCTCCAAAGAGC |
| BD-SDE19-F | CATATGGCCATGGAGGCCGAATTCATGGCCAATGAGCACTCTTCTGT |
| BD-SDE19-R | CGGCCGCTGCAGGTCGACGGATCCTCAATTGTTTAAGCCTCCAAAG |
| AD19-3-F | ATGGCCATGGAGGCCAGTGAATTCATGCAGATCTTCGTGAAAACCCT |
| AD19-3-R | CTGCAGCTCGAGCTCGATGGATCCTCAGAAACCACCACCTCGGAG |
| AD19-4-F | ATGGCCATGGAGGCCAGTGAATTCATGACGGTAACGCCGAACATCT |
| AD19-4-R | CTGCAGCTCGAGCTCGATGGATCCTTAGACTTGGATCTCAACAGGCCATCT |
| AD19-12-F | ATGGCCATGGAGGCCAGTGAATTCATGAGTACTGGAGGCGAGAAAGGT |
| AD19-12-R | CTGCAGCTCGAGCTCGATGGATCCCTAGGCCCCATCTGCTAATGTTCTC |
| AD-Sec12-F | ATGGCCATGGAGGCCAGTGAATTCATGGGTAGCAGGAGCAGGAAT |
| AD-Sec12-R | CTGCAGCTCGAGCTCGATGGATCCCTAAGGAATGATTCCTTTTGCCTTCACAAA |
| AD-SEC12N-R | CTGCAGCTCGAGCTCGATGTTAGGAGAGGGAATTGGAGGCGA |
| AD-WD-F | ATGGCCATGGAGGCCAGTGAATTCGATCAGCCGGTGGCTGAGCT |
| AD-WD-R | CTGCAGCTCGAGCTCGATGTTATACCGTGACCCTTACACTTGA |
| AD-WD1-R | CTGCAGCTCGAGCTCGATGTTAGGCTGAAGAAGCTAGATCCC |
| AD-EDR2-F | ATGGCCATGGAGGCCAGTGAATTCATGGAAACGACGTCGTCGGTAAAG |
| AD-EDR2-R | CTGCAGCTCGAGCTCGATGGATCCTCAAACCTCCAATGGTACAGCAGC |
| G2-SDE19-F | GATCTTGGGCCCAGGCCTACTAGTATGGCCAATGAGCACTCTTCTGT |
| G2-SDE19-R | CTACCCGGGAGCGGTACCCTCGAGTCAATTGTTTAAGCCTCCAAAG |
| G4-Sec12-F | TACGCTGGGCCCAGGCCTACTAGTATGGGTAGCAGGAGCAGGAAT |

| G4-Sec12-R | | CTACCCGGGAGCGGTACCCTCGAGCTAAGGAATGATTCCTTTTGCCTTCACAAA | |
| --- | --- | --- | --- |
| G4-EDR2-F | | TACGCTGGGCCCAGGCCTACTAGTATGGAAACGACGTCGTCGGTAAAG | |
| G4-EDR2-R | | CTACCCGGGAGCGGTACCCTCGAGTCAAACCTCCAATGGTACAGCAGC | |
| Myc-SDE19-F | | GAGGAAGACTTGAACGGTGAATTCATGGCCAATGAGCACTCTTCTGT | |
| Myc-SDE19-R | | ATCTCATTAAAGCAGGACTCTAGATCAATTGTTTAAGCCTCCAAAG | |
| GFP-Sec12-F | | AGAACACGGGGGACGAGCTCATGGGTAGCAGGAGCAGGAAT | |
| GFP-Sec12-R | | ACCATGGTGTCGACTCTAGAAGGAATGATTCCTTTTGCCTTCACAAAGTA | |
| GFP-EDR2-F | | AGAACACGGGGGACGAGCTCATGGAAACGACGTCGTCGGTAAAG | |
| GFP-EDR2-R | | ACCATGGTGTCGACTCTAGAAACCTCCAATGGTACAGCAGCA | |
| 2300PR1-F | | AGAACACGGGGGACGAGCTCATGGGATTTGTTCTCTTTTCCC | |
| 2300PR1-R | | ACCATGGTGTCGACTCTAGAGTATGGACTTTGGCCAATAAC | |
| 2300P69B-F | | AGAACACGGGGGACGAGCTCatgggattattgaaaatccttcttg | |
| 2300 P69B-R | | ACCATGGTGTCGACTCTAGAggcagacacaactgcaattg | |
| 2300RCR3-F | | AGAACACGGGGGACGAGCTCatggctatgaaagttgatttgatg | |
| 2300RCR3-R | | ACCATGGTGTCGACTCTAGAcgctatgtttggataagaagaca | |
| 2300GmGIP1-F | | AGAACACGGGGGACGAGCTCatgcctcctcctcttccatc | |
| 2300GmGIP1-R | | ACCATGGTGTCGACTCTAGAtttaattccatttgcaaaattattgac | |
| 2300PDF1.2-F | | | AGAACACGGGGGACGAGCTCatggctaagtttgcttccatc |
| 2300PDF1.2-R | ACCATGGTGTCGACTCTAGAacatgggacgtaacagataca | | |
| V-Sec12-F | tgagtaaggttaccgaattcATTCTCGCCAGTTAATGATGAGAATC | | |
| V-Sec12-R | gtgagctcggtaccggatccAGCAAAGCCCTCGAATCTTCC | | |
| NbSec12-qF | AGGAGGTGGAGAAGGGAATAG | | |

| NbSec12-qR | ACCACCAGTTCCTTCCTTTG |
| --- | --- |
| NbEF1a_F | TCTTCCACTTCAGGACGTTTAC |
| NbEF1a_R | TCCAAAGGTCACAACCATACC |
