# Supplementary figures and images for: SDE19, a SEC-dependent effector from ‘Candidatus Liberibacter asiaticus’ suppresses plant immunity and targets Citrus sinensis Sec12 to interfere with vesicle trafficking
Source: PLoS Pathog. 2024 Sep 10;20(9):e1012542. doi: 10.1371/journal.ppat.1012542 (PMC11414923; doi:10.1371/journal.ppat.1012542)

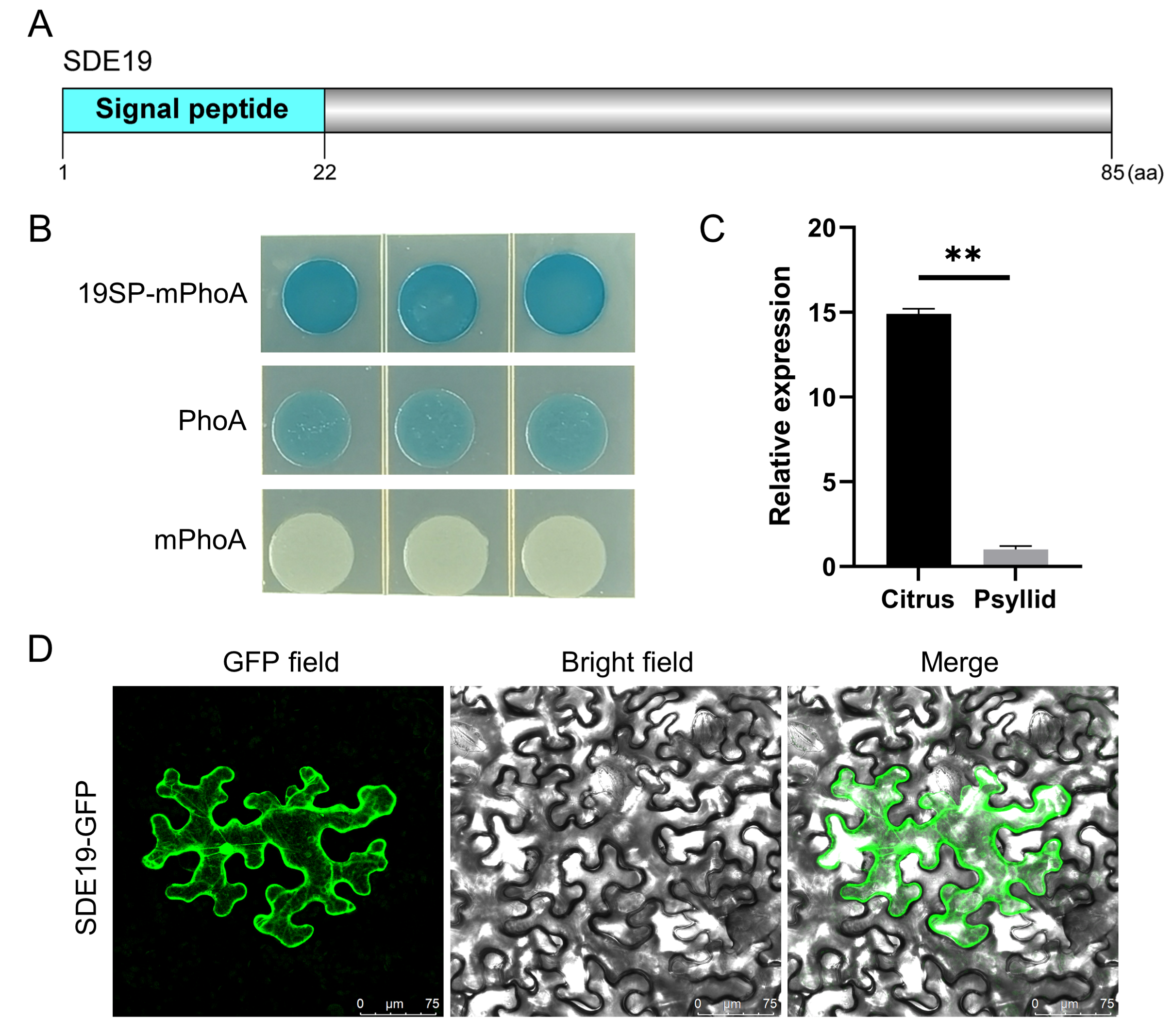

Supplement: S1 Fig — (A) Sequence analysis of SDE19, (B) The signal peptide of SDE19 is capable of directing protein secretion. The signal peptide of SDE19 was fused with mature PhoA to assess alkaline phosphatase activity in Escherichia coli. Full length phoA was used as the positive control, while mature PhoA without SP served as the negative control for non-secretion. (C) Relative expression of SDE19 in CLas-infected Citrus sinensis and Asian citrus psyllids by reverse transcription-quantitative PCR (RT-qPCR). gyrB was used as an endogenous control. Bars represent the standard deviation (SD) of the means, double asterisks indicate P value less than 0.01 using Student’s t test. (D) Subcellular localization analysis of SDE19-GFP in Nicotiana benthamiana. (TIF) [file ppat.1012542.s006.tif]

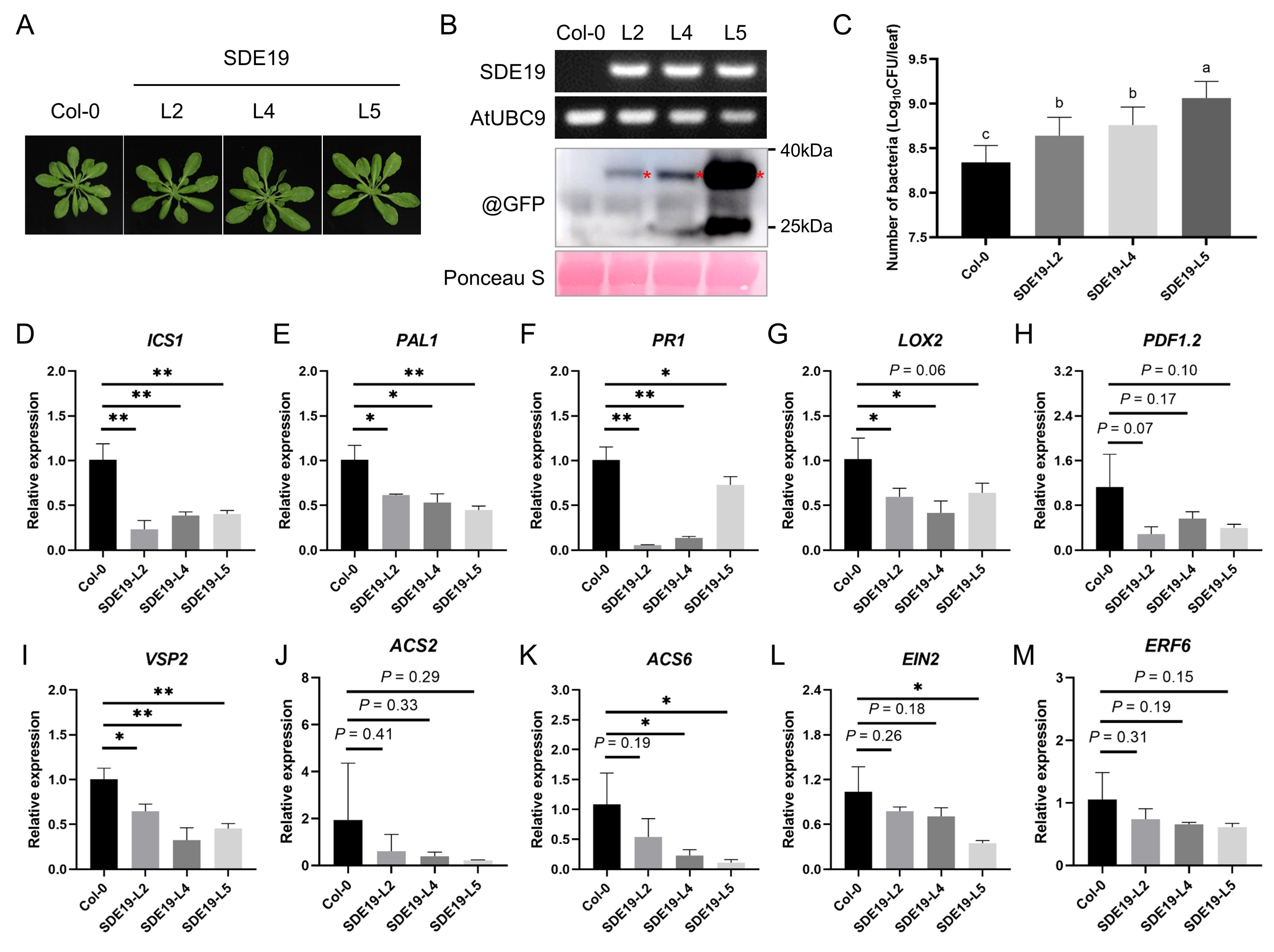

Supplement: S2 Fig — (A) Growth phenotype of SDE19 transgenic A. thaliana lines. (B) Verification of SDE19 expression using semi-quantitative PCR and western blot. AtUBC9 was used as the internal reference gene. The asterisks (*) represent the protein bands of SDE19-GFP, which were detected by anti-GFP antibodies. (C) SDE19 promotes the colonization of Pseudomonas syringae pv. tomato (Pst) strain DC3000 in transgenic A. thaliana plants. Mature leaves of transgenic A. thaliana lines and wild-type Col-0 were inoculated with Pst DC3000 cell suspensions. Bacterial colonization was determined as colony forming units (CFU/ leaf) at 3 days post-inoculation. Bars represent the standard deviation (SD) of the means from 8 samples of 16 leaves. Different letters (a, b, and c) above the bar indicate statistically significant differences (P < 0.05) based on a one-way ANOVA followed by turkey’s multiple range test. Similar results were obtained from three independent experiments. (D-M) The relative expression of ACS2, ACS6, EIN2, ERF6, ICS1, LOX2, PAL1, PDF1.2, PR1 and VSP2, in SDE19 transgenic A. thaliana at 36 hours post-inoculation of Pseudomonas syringae pv. tomato DC3000 was detected by reverse transcription-quantitative PCR. Bars represent the standard deviation (SD) of the means, an asterisk indicates P value less than 0.05, and double asterisks indicate P value less than 0.01 using Student’s t test. AtUBC9 was used as the internal reference gene. (TIF) [file ppat.1012542.s007.tif]

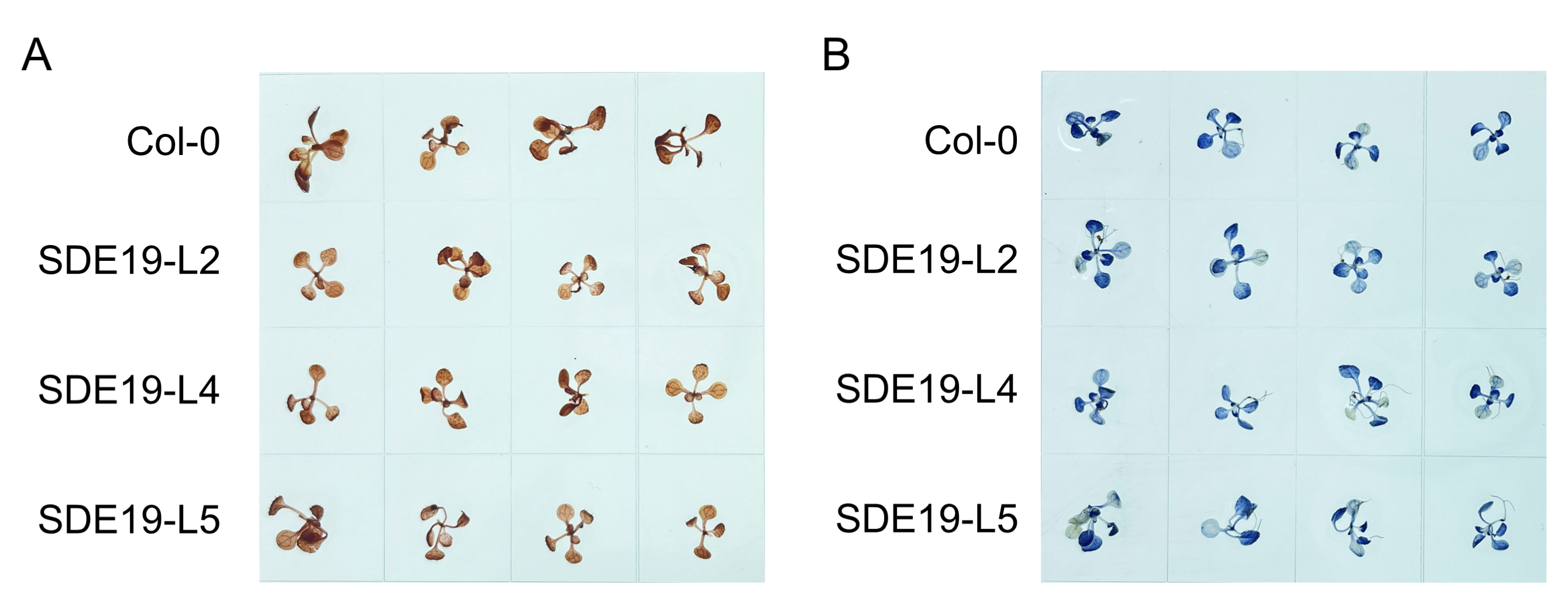

Supplement: S3 Fig — (A) DAB staining of hydrogen peroxide (H2O2) accumulation in SDE19 transgenic A. thaliana and wild-type Col-0. (B) NBT staining of superoxide in SDE19 transgenic A. thaliana and Col-0. (TIF) [file ppat.1012542.s008.tif]

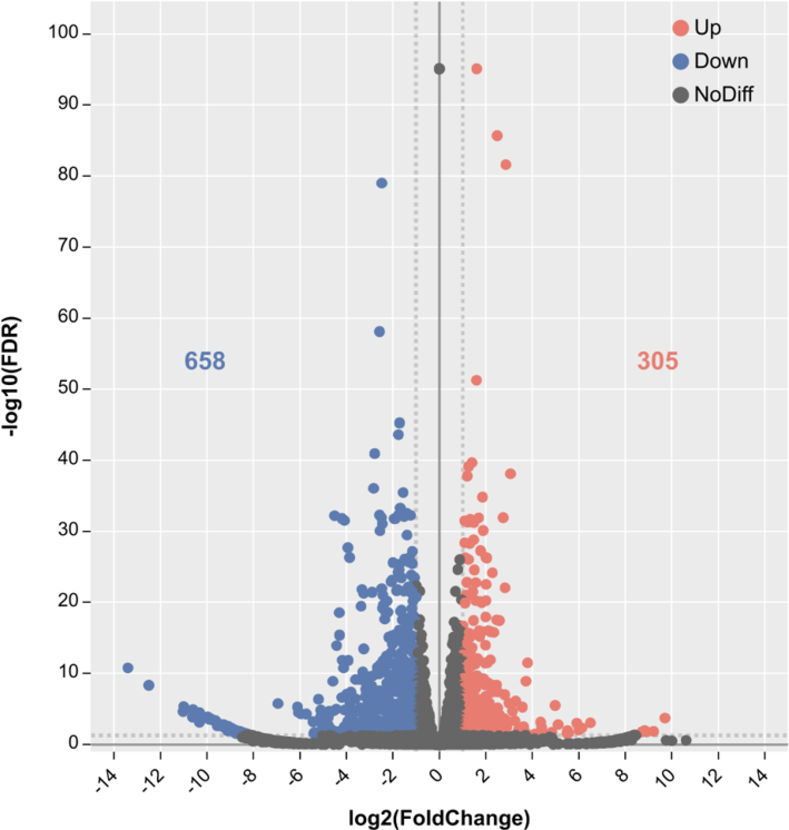

Supplement: S4 Fig — The blue number represents the quantity of down-regulated genes, while the red number represents the quantity of up-regulated genes. (TIF) [file ppat.1012542.s009.tif]

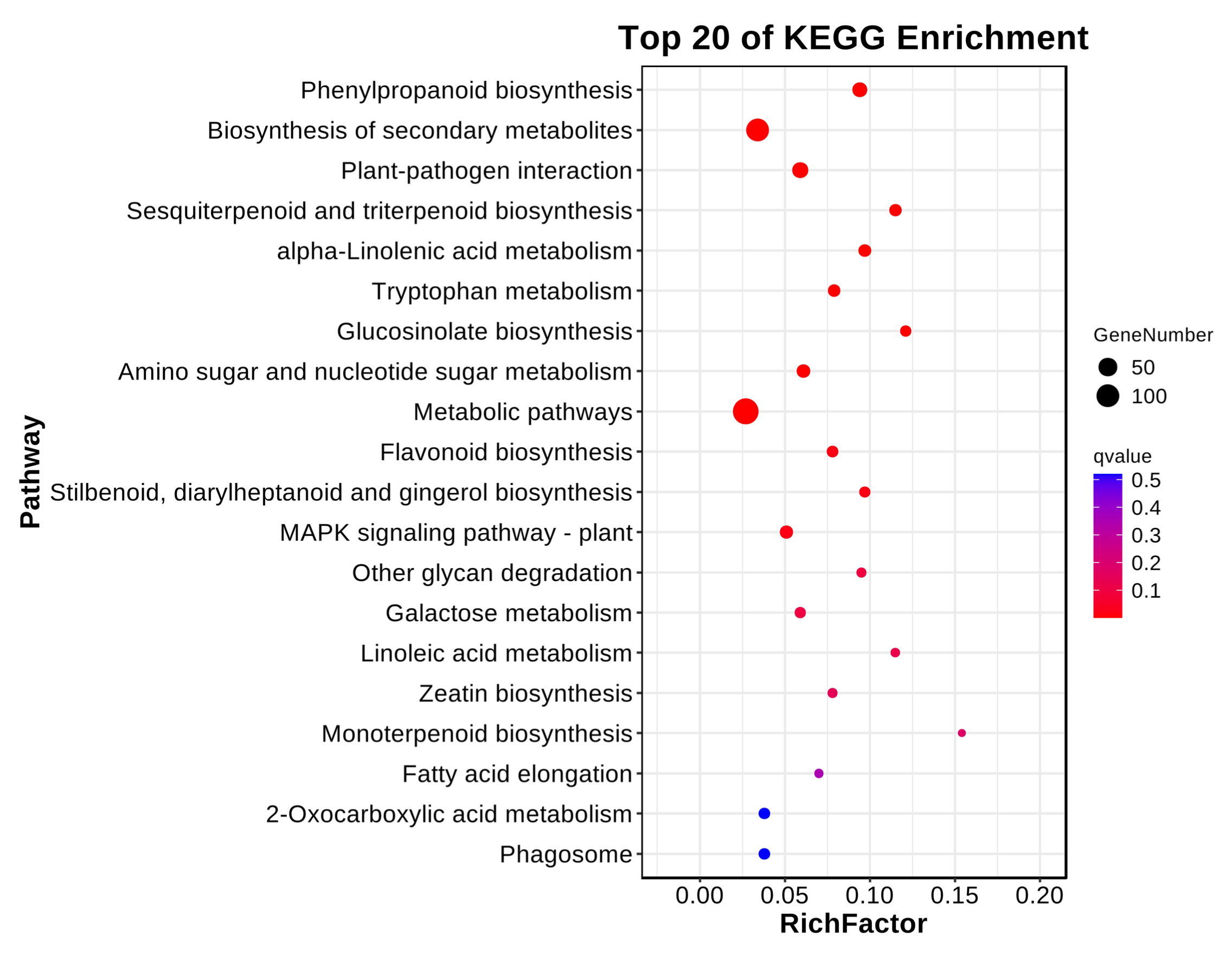

Supplement: S5 Fig — (TIF) [file ppat.1012542.s010.tif]

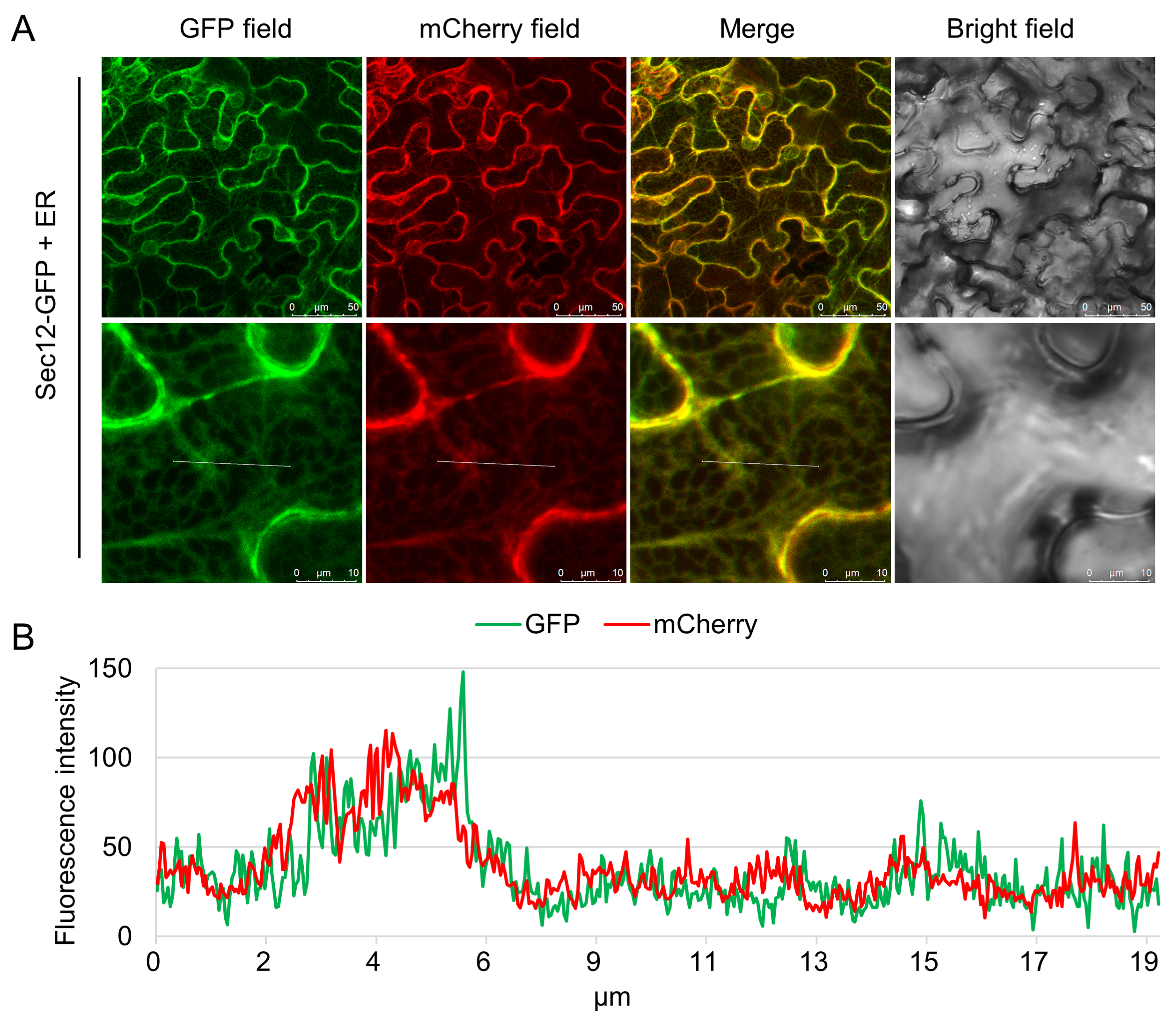

Supplement: S6 Fig — (A) Confocal images of Sec12-GFP and the mCherry labeled endoplasmic reticulum marker were taken at 2 days post-inoculation. The bottom row shows partially enlarged micrographs of the ER network. (B) A profile of the fluorescence intensities of GFP and mCherry aligned with the white line in (A). (TIF) [file ppat.1012542.s011.tif]

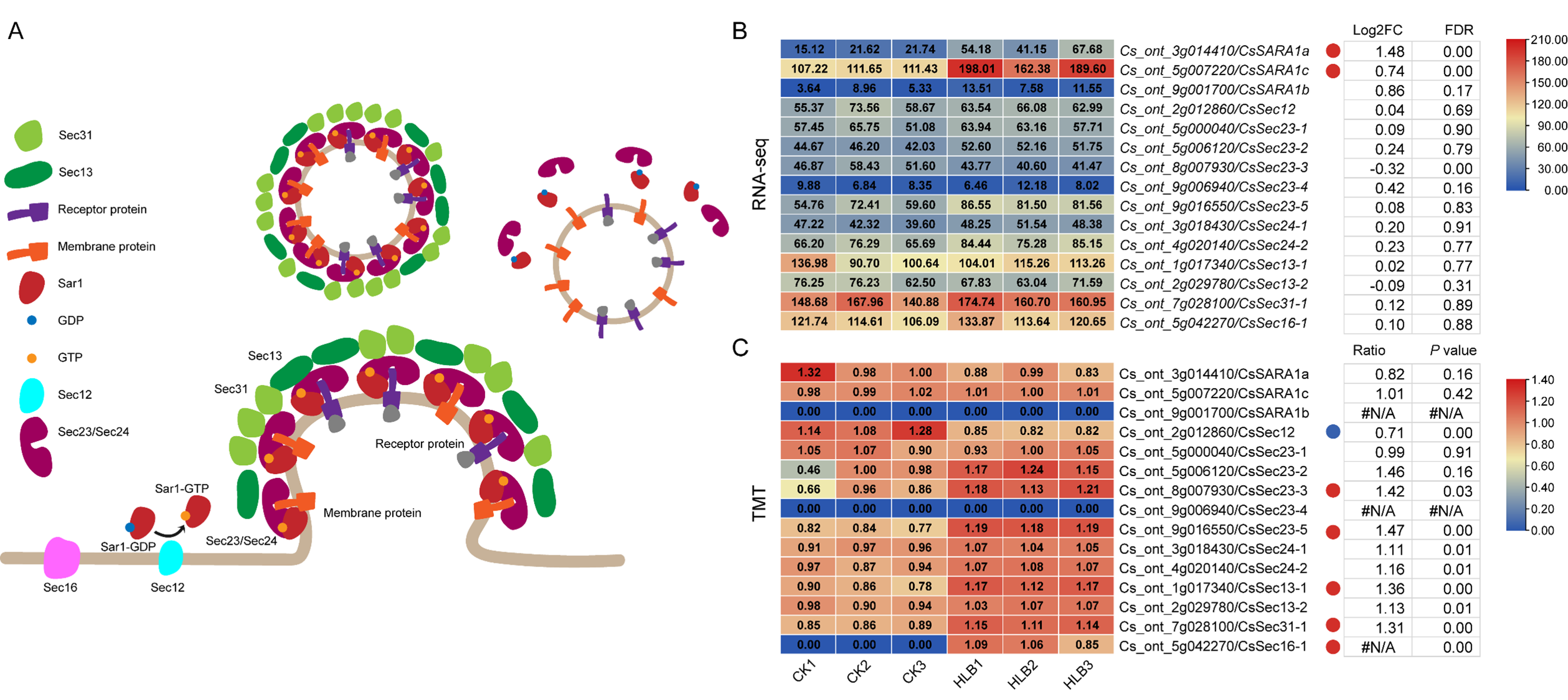

Supplement: S7 Fig — (A) Schematic diagram of the COPII-coated vesicles assembly process adapted from [36]. (B) RNA-seq analysis revealed differential expressed genes involved in the vesicle trafficking pathway during CLas infection. SARA represents SECRETION-ASSOCIATED RAS 1. (C) Quantitative proteome analysis revealed differential accumulation of proteins involved in the vesicle trafficking pathway during CLas infection. The red dots represent up-regulated genes or proteins, while the blue dots represent down-regulated proteins. FC stands for Fold change, while #N/A indicates that the protein was not detected during mass spectrum analysis. (TIF) [file ppat.1012542.s012.tif]
